# Come rain or come shine: environmental effects on the infective stages of *Sparicotyle chrysophrii*, a key pathogen in Mediterranean aquaculture

**DOI:** 10.1186/s13071-018-3139-3

**Published:** 2018-10-25

**Authors:** Mar Villar-Torres, Francisco Esteban Montero, Juan Antonio Raga, Aigües Repullés-Albelda

**Affiliations:** Cavanilles Institute of Biodiversity and Evolutionary Biology, Science Park, University of Valencia, Paterna, Valencia Spain

**Keywords:** Monogenea, Platyhelminthes, Free-living stages, *Sparus aurata*, Climate change, Abiotic factors, Temperature, pH, Photoperiod, Salinity

## Abstract

**Background:**

Evidence concerning the environmental influence on monogenean transmission and infection processes is widely accepted, although only the effects of a limited number of abiotic factors on particular monogenean species have been explored. The current context of climate change calls for further research both on this subject, and also that concerning monogenean hosts, especially in aquaculture.

**Methods:**

In this study, four experiments were used to assess the response of the infective stages of *Sparicotyle chrysophrii*, a pathogenic monogenean from gilthead sea bream (*Sparus aurata*) cultures in the Mediterranean, to variations of temperature (from 10 °C to 30 °C), pH (7.0 and 7.9), photoperiod (LD 12:12, LD 0:24 and LD 24:0) and salinity (from 27 ppt to 47 ppt).

**Results:**

Thermal variations cause the strongest responses among the infective stages of *S. chrysophrii*, which reduced development and survival times as temperature increased. The optimal thermal range for maximum hatching success was found between 14 and 22 °C, whereas temperatures of 10 and 30 °C probably represent biological thermal limits. Reductions of development time and hatching rates were recorded at the lowest pH level, but hatching success remained above 50%, suggesting a certain degree of tolerance to slight pH variations. Photoperiod acts as an environmental cue synchronising the circadian hatching rhythm of *S. chrysophrii* with the first four hours of darkness. Response to a wide range of salinities was negligible, suggesting a high tolerance to variations of this abiotic factor.

**Conclusions:**

Larval development and hatching of *S. chrysophrii* are modulated according to environmental factors, mainly temperature, thus parasite-host coordination and successful infections are enhanced. Therefore, abiotic factors should be broadly considered to design treatments against this monogenean. The high tolerance to the predicted environmental variations over the next century reported for gilthead sea bream and herein exposed for *S. chrysophrii* suggests that neither will be notably affected by climate change in the western Mediterranean region.

**Electronic supplementary material:**

The online version of this article (10.1186/s13071-018-3139-3) contains supplementary material, which is available to authorized users.

## Background

Traditionally, the host-parasite relationship is associated with the balance that both organisms are able to maintain, taking into account the pathological effects of the parasite on its host as well as the host’s defence and recovery mechanisms [1]. Additionally, hosts and parasites are directly and indirectly exposed to a changing environment that affects them and modifies the stability of their relationship [2–4]. Focusing on parasites, the influence of the environment is especially relevant for free-living stages, although in the case of ectoparasites as monogeneans every stage of their life-cycle is directly exposed to variable water conditions [2, 5]. Since the infective stages, eggs and oncomiracidia, have a significant role on monogenean transmission and dispersion, the effects of a changing environment can alter monogenean infection dynamics [6–8]. Therefore, analysing the responses of these infective stages to the environmental variability is required, particularly in the current context of climate change where relatively rapid variations in abiotic conditions are predicted [9].

Of the abiotic factors expected to be altered by climate change, i.e. temperature, pH, salinity, and certain aspects relative to light (intensity and radiation) [9], only temperature, salinity and sporadically light (intensity and photoperiod) are reported to affect the free-living stages of monogeneans, including egg hatching, development, larval survival and swimming behaviour of several species [10–12]. Temperature stands out among these factors as it is reported to affect hatching (period and success), incubation period, larval longevity and swimming behaviours of many monogenean species [6, 7, 10, 11, 13–15]. To our knowledge, no studies have been conducted to determine whether pH modifies biological features of the eggs and larvae of monogeneans, although water acidification has been reported to influence availability, longevity and survival in the infective stages of other platyhelminths, such as trematodes [16, 17]. Therefore, the effect of pH on monogenean eggs and larvae should be further studied. Despite the effects of climate change on photoperiod and light intensity being marginal, both are known to play key roles in synchronising monogenean hatching rhythms ([12] and references therein). However, the influence of photoperiod on other parameters, such as hatching success, oncomiracidium development and larval behaviour, has only been explored in a small number of monogenean species e.g. *Entobdella soleae* [18] and *Neoheterocotyle rhinobatidis* [19]. Studies dealing with salinity assess the therapeutic activity of hyposaline conditions on egg hatching and oncomiracidium survival in marine monogeneans [20–23]; however, there is minimal research on the effect of hypersaline conditions on monogeneans, except for isolated studies on *Benedenia seriolae* [21], *Dendromonocotyle pipinna* [22] and *Neobenedenia* spp. [15], which also examine other developmental and behavioural variables.

The effects of biotic and abiotic factors on free-living stages of monogeneans are especially relevant in aquaculture, particularly in temperate environments where seasonal variations can determine monogenean infection dynamics. This could be the case of *Sparicotyle chrysophrii* (Van Beneden & Hesse, 1863), a pathogenic parasite found on wild and cultured gilthead sea bream (*Sparus aurata*) [24–26], which is the most important fish species in Mediterranean aquaculture [27, 28]. Indeed, in this region, gilthead sea bream cultures are exposed to seasonal variations, which have been associated with the highest infection levels of *S. chrysophrii* recorded during spring (from March to June) and early summer (June and July) [24, 29–31]. However, abiotic factors underlying infection dynamics and their influence on infective stages of *S. chrysophrii* remains unexplored, which hampers management of this parasite in aquaculture.

Recurrent infections of *S*. *chrysophrii* may lead to epizootic episodes and cause major losses in gilthead sea bream cultures [24–26]. In addition, reinfection is likely since common treatments are ineffective against monogenean eggs [32]. Therefore, to determine the appropriate treatment frequency, the time between parasite removal and the emergence of new oncomiracidia (development time) must be determined [33, 34]. The chronology of *S. chrysophrii* development was described in two previous studies, but the authors only used one set of environmental conditions, i.e., at 22 °C [35] and 20 °C, LD12:12 [33, 34]; therefore, further study is required. This study deals with the examination of the effects of temperature, pH, photoperiod and salinity variations on the biology of the infective stages of *S. chrysophrii*, considering climate change projections for the Mediterranean Sea. We also aim to find suitable treatment schedules to manage *S. chrysophrii* infections based on different environmental conditions in aquaculture facilities.

## Methods

### Parasite collection and experimental design

Four experimental studies were conducted using *S. chrysophrii* eggs and larvae to address the influence of four abiotic factors, i.e. temperature, pH, photoperiod and salinity combined with temperature, on development, hatching, swimming behaviour and larvae survival. *S*. *chrysophrii* eggs (*n* = 4800) were obtained from the adult parasites of 41 recently dead gilthead sea breams (standard length: 10.5–24.5 cm; weight: 32.4–329.0 g), which were collected from a fish farm off the Spanish Levantine coast (western Mediterranean) (37°30'N, 1°37'W to 40°31'N, 0°30'E) during summer 2014.

Fish were immediately dissected and gills were isolated and examined using a Leica MZ APO stereomicroscope (Wetzlar, Germany; 8–100×) with transmitted light. Gravid parasites were removed, collected and deposited alive in a Petri dish with sea water. Next, based on the methods of Repullés-Albelda et al. [34], gravid *S. chrysophrii* were mechanically disrupted to obtain the eggs. Once collected, eggs were reviewed to avoid those empty or deformed and their developmental degree was checked. Only those full of vitelline material with undistinguishable embryo were included in the experiment (see Fig. 1a, b in [34]). Selected eggs were cleaned with sterilized sea water washes. Thereafter, the eggs were randomly mixed and placed into separate wells with 3 ml of daily-renewed sea water each. These wells were reared in environmental chambers (Ing. Clima, model CIR-S 250, temperature control ± 0.1 °C), with an 8 W fluorescent tube fitted to the chamber ceiling, at different abiotic conditions as per the four experiments described below. The abiotic factors were analysed by levels with three replicates of 100 eggs each, except for salinity with 50 eggs per replicate.

Experiment 1: Assessment of temperature effect. Eggs were reared in sea water at 37 ppt, pH 7.9 (± 0.1), subjected to 12 h periods of alternating artificial light and darkness (LD 12:12) and incubated separately at 10, 14, 18, 22, 26 and 30 °C. Three additional wells with 100 eggs were incubated at 10 °C to guarantee the reliability of the low hatching success recorded at this temperature. Therefore, data on hatching success at 10 °C also include the results of these additional replicates reported in tables. Moreover, while statistical analysis of development, swimming and survival variables were performed using those replicates with emerged larvae, hatching success was analysed with the first set of data by temperature. The temperature range was selected to represent common (14–26 °C) and extreme temperatures (10 and 30 °C) registered in the western Mediterranean region per data provided by the Spanish agency, Puertos del Estado [36].

Experiment 2: Assessment of pH effect. Eggs were maintained in sea water at 37 ppt, LD 12:12, 22 °C at two pH levels: pH 7.0 (± 0.1) and 7.9 (± 0.1), which was performed in the previous experiment. Sea water pH was measured using a Crison pH-meter (Basic 20, Barcelona, Spain) and adjusted to pH 7.0 (± 0.1) by adding 0.1 M HCl to sea water. The pH levels were selected to represent the average pH of the Mediterranean Sea and decreased sea water pH including climate change predictions [37].

Experiment 3: Assessment of photoperiod effect. Eggs were incubated in 37 ppt, pH 7.9 (± 0.1), 22 °C sea water under different light regimes: alternate (LD 12:12) and constant light conditions (LD 0:24 and 24:0).

Experiment 4: Assessment of combined salinity and temperature effects. Eggs were maintained in sea water at pH 7.9 (± 0.1) and LD 12:12 at five salinity conditions (27, 36, 37, 38 and 47 ppt), and two temperatures (18 and 22 °C). Salinities between 36 and 38 ppt were chosen to represent the water salinity range in the western Mediterranean region. Additionally, salinities of 27 and 47 ppt were selected to determine the influence of extreme salinities on the infective stages of *S. chrysophrii*. Saline solutions were made by adding marine salt or distilled water to 37 ppt filtered sea water and adjusted using a refractometer (Milwaukee MR 128, Rocky Mount, USA). Levels for both factors were selected to represent those normally found in spring [36], when epizootics of this parasite are known to occur in the western Mediterranean [24, 29, 30].

Wells from the four experiments were introduced simultaneously at 19:00 into the different environmental chambers, coinciding with the start of the 12 h dark period under the alternating light conditions. This moment was established as time 0 for all experiments, since the time elapsed between egg collection and the start of incubation in the environmental chambers was considered negligible for analyses. Eggs were initially monitored every 12 h on concave slides using a Leica DMR light microscope (Wetzlar, Germany; 100–1000×) until the first detection of eye-spots, which was considered to represent near-hatching according to previous studies [34]. The time until the first eyed-egg was recorded for each factor level in the four experiments. Thereafter, the monitoring frequency increased to 4 h intervals until the last egg hatched. Six observation periods were established for every 24 h: three periods under dark conditions [(1) 19:00–23:00 h; (2) 23:00–3:00 h;  and (3) 3:00–7:00 h] and three under light conditions [(4) 7:00–11:00 h; (5) 11:00–15:00 h; and (6) 15:00–19:00 h]. Egg monitoring stopped when hatching ceased for at least 48 h at each factor level. Hereafter, oncomiracidium development will be referred to as embryonic development inside the egg and larval development after egg hatching.

Embryonic development and hatching analyses were performed using four variables for every observation: total number hatched; light condition (light/dark); observation period (1 to 6); and individual incubation period, defined as time to egg hatching in hours. To compare larval emergence after first hatching, Day 1 was assigned to the first day with hatchings (from 19:00 to 16:00 h) at each factor level. Moreover, hatching period and hatching success were calculated per replicate. Hatching period was established as the elapsed time between the first and last hatching, and hatching success was defined as the ratio of empty eggs with an open operculum and the total number of eggs. In addition, the hatching peak was defined as the observation moment when the maximum number of hatchings occurred in each experiment.

The oncomiracidia that emerged from these eggs (*n* = 3509) were used for development follow-up. Larvae were collected after hatching and individually arranged in new wells with 400 μl of sea water using a micropipette. They were maintained under the same environmental conditions as their egg incubation. Wells were covered with coverslips to prevent oncomiracidia death when they get trapped in the water surface film (see [34]). In this case, sea water was not renewed to avoid abrupt modification of the oncomiracidia environment. Based on previous studies [34], oncomiracidial swimming behaviour was verified as normal when larva exhibited variations on swimming speed and trajectory. Swimming and survival of each oncomiracidium were recorded every 4 h. From these records, the ratio between swimming and survival period (swimming ratio) was calculated. A survival period of 0 h was established for those oncomiracidia that emerged from the egg but died before being collected. Oncomiracidia were considered dead when they did not respond to mechanical stimuli. All observations were recorded using a Leica MZ APO stereomicroscope (8–100×).

### Statistical analyses

Nonparametric tests (Mann-Whitney U-test and Kruskal-Wallis H-test) were performed to examine differences in the incubation period, hatching success and swimming ratio between temperatures, pH levels and light regimes (Experiments 1, 2 and 3). In those cases where significant differences were detected, Dunn’s *post-hoc* tests were conducted for pairwise comparisons. Generalised linear models (GLMs) were performed in Experiment 4 to assess the effect of environmental factors on incubation period, hatching success and swimming ratio using different temperature and salinity combinations as explanatory variables. Error types were adapted to the models used; Poisson error with a log link function was used for the incubation period and swimming ratio, whereas a binomial error with logit link function was used for hatching success. A set of alternative models with different combinations of the two explanatory variables were conducted for each response variable using a stepwise process. Akaike information criterion (AIC) was used as the model selection criteria and the significance of effects was determined with likelihood-ratio tests. In accordance to Burnham & Anderson [38], models within two Akaike units from the best model were considered equivalents. Kruskal-Wallis test were also conducted at every experiment to test for differences in developmental and hatching variables between replicates. Results on statistical tests are only detailed for non-consistent replicates.

The number of hatchings was analysed for every level within each environmental factor by using GLMs with observation period (1−6), light condition (light/dark) and number of days (from first to last night of hatching) added as explanatory variables. Data were treated as a count, thus a Poisson error with a log link function was implemented.

Finally, survival analyses were performed to explore the influences of temperature, pH, photoperiod and salinity on oncomiracidia survival in the four experiments. Survival function curves were created using the Kaplan-Meier estimator and differences between the levels of each factor were assessed using a log-rank test. Likewise, to model hazard functions and determine the effects of these factors on oncomiracidia survival, Cox’s proportional hazard models were conducted. As a single abiotic factor was evaluated in Experiments 1, 2 and 3, corresponding to temperature, pH and light, respectively, a single Cox model was fitted for each factor. However, since both salinity and temperature were included in Experiment 4, several models were developed for different combinations between both factors. Model selection was also based on the Akaike information criterion. Proportional hazard assumption of Cox models was tested using the R function “cox.zph()”. All statistical analyses were performed using the R packages: *stats*, *dunn.test* [39] and *survival* [40] in R v.3.1.2 software [41]. *P*-values lower than 0.05 were considered statistically significant.

## Results

### Experiment 1: Assessment of the temperature effects

Embryonic development of *S. chrysophrii* was affected by water temperature, with shorter developmental times occurring at increasing temperatures. The first developmental event recorded, eye-spot detection, occurred earlier at higher temperatures (after 96 h at 26 °C and 22 °C, 108 h at 18 °C and 168 h at 14 °C). This trend was also found among extreme temperatures with eye-spot detection ranging from 108 h at 30 °C to 384 h at 10 °C.

**Fig. 1:**
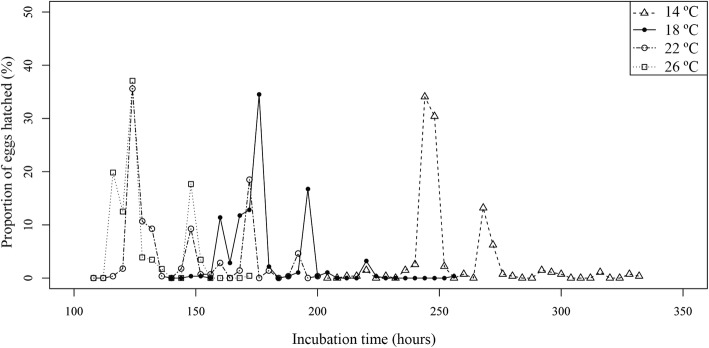
Proportion of hatched *Sparicotyle chrysophrii* eggs at each observation moment, incubated at four temperatures

Incubation period was also influenced by water temperature, being shorter at higher temperatures (Table 1 and Fig. 1). Differences between incubation periods were statistically significant among temperatures (*P* < 0.001). *Post-hoc* analyses allowed distinguishing between five groups of incubation periods associated with different temperatures: 10 °C, 14 °C, 18 °C, 22 °C/30 °C and 26 °C (*P* < 0.05). The mean incubation period decreased at common temperatures from 14 °C to 22 °C/26 °C. However, at extreme temperatures this trend was only accomplished for 10 °C, while at 30 °C mean incubation period was longer than at 26 °C (Table 1). Results by replicates were mostly in accordance with this pattern (Additional file 1: Table S1), although most of the replicates within each temperature were significantly different (*P* < 0.05). Similarly, hatching periods were shorter at higher temperatures, but data by replicates partially overlapped among consecutive levels (Table 1 and Additional file 1: Table S1). Hatching peaks were generally detected during the first third of the hatching period at every temperature (corresponding to 20 h at 30 °C, 17 h at 26 °C, 25 h at 22 °C, 32 h at 18 °C, 36 h at 14 °C and 32 h at 10 °C) (Fig. 1) and for most replicates (Additional file 1: Table S1). Mean hatching success was also affected by temperature, with many oncomiracidia emerging at 14 °C, 18 °C and 22 °C, decreasing slightly at 26 °C and declining markedly at the highest and the lowest temperatures tested (Table 1). Differences in hatching success were significant among temperatures (*P* < 0.05). Two groups of temperatures were significantly different after *post-hoc* analysis: one included those eggs incubated at 10 °C, 26 °C and 30 °C and the other those at 14 °C, 18 °C, 22 °C and 26 °C (*P* < 0.05).

**Table 1:** Parameters of embryonic development of *Sparicotyle chrysophrii* by environmental factor

Environmental factor	Factor level	Incubation period (h)	Hatching period (h)	Hatching peak^a^ (h)	Hatching success (%)
		Mean ± SD (Range)			
Temperature (± 0.1 °C)	10	995.5 ± 23.6 (948–1044)^b^	96^b^	988^b^	7.3^c^
	14	253.4 ± 17.3 (212–332)	120	248	91.0
	18	178.1 ± 14.8 (148–256)	108	176	93.3
	22	143.1 ± 22.5 (116–200)	84	124	93.7
	26	128.0 ± 12.0 (116–172)	56	124	77.3
	30	148.8 ± 20.8 (124–184)	60	148	6.0
pH (± 0.1)	7.0	150.0 ± 9.0 (144–176)	32	144/148	56.0
	7.9	143.1 ± 22.5 (116–200)	84	124	93.7
Light regime (light:darkness)	12:12	147.4 ± 25.6 (120–200)	80	124	93.0
	0:24	125.1 ± 11.3 (112–232)	120	124	89.3
	24:0	141.4 ± 23.4 (108–268)	160	140	88.0
Salinity(ppt)-Temperature (± 0.1 °C)	27−18	191.7 ± 13.6 (152–220)	68	196	89.3
	36−18	169.6 ± 15.3 (140–268)	128	168	85.3
	37−18	172.7 ± 18.2 (140–228)	88	172	64.7
	38−18	179.5 ± 12.7 (168–220)	52	168	80.7
	47−18	218.7 ± 11.5 (180–244)	64	220	74.6
	27−22	141.8 ± 11.5 (120–168)	148	148	85.3
	36−22	129.2 ± 9.3 (108–156)	48	124	74.7
	37−22	138.3 ± 16.3 (124–196)	72	128	82.0
	38−22	135.0 ± 16.7 (124–228)	104	128	69.0
	47−22	182.2 ± 12.7 (148–204)	56	176	64.7

^a^Hatching peak; moment when the highest number hatchings was registered

^b^Includes replicates with emerged eggs (*n* = 3)

^c^Includes all replicates (*n* = 6)

**Fig. 2:**
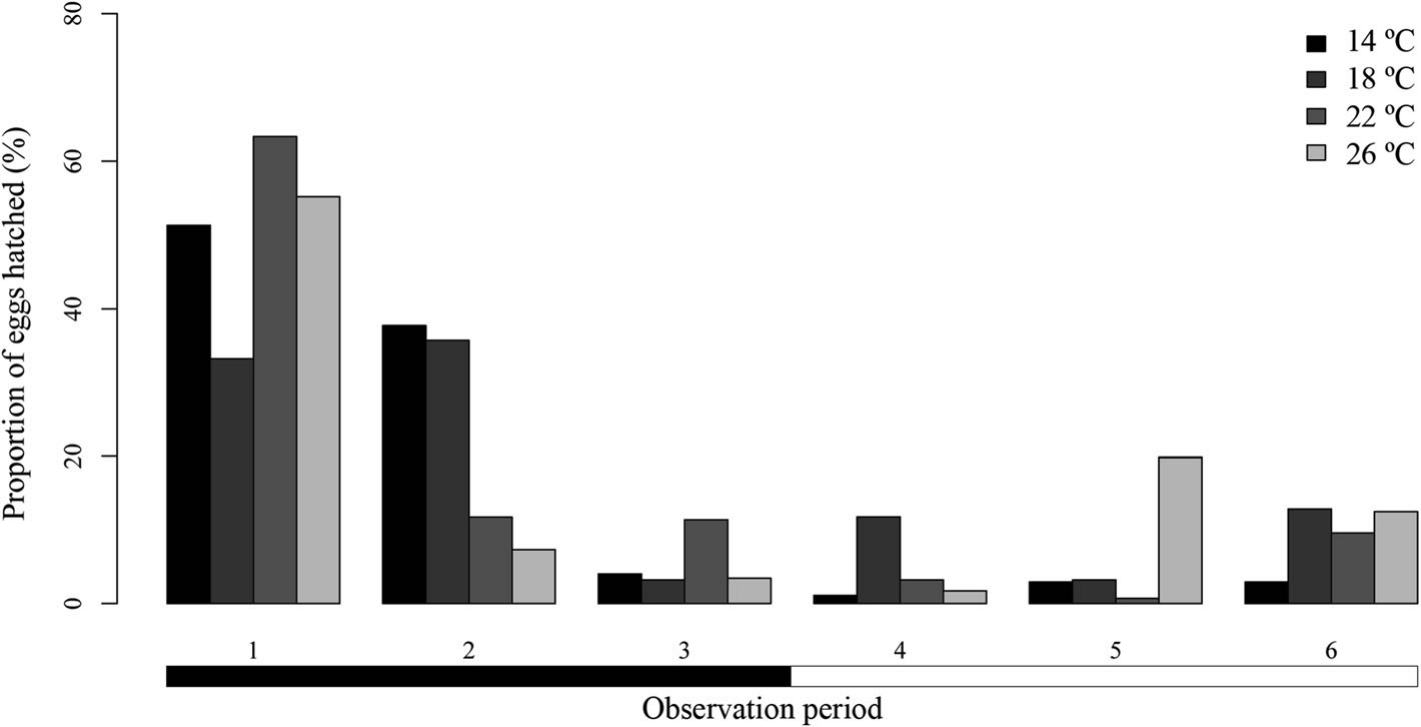
Proportion of hatched *Sparicotyle chrysophrii* eggs for each observation period, incubated at four temperatures. Horizontal bars below the chart indicate darkness (black) and light (white)

More than the 66% of the eggs hatched during the dark periods for all temperatures and most of the replicates (Fig. 2). Based on the models, the variation in the number of hatchings was significantly affected by number of day (*P* < 0.001) and observation period (*P* < 0.001). The best models retained these two factors for all temperatures: 14 °C (AIC = 201.7; 88% of explained variation); 18 °C (AIC = 46.4; 47% of explained variation); 22 °C (AIC = 189.8; 86% of explained variation); and 26 °C (AIC = 342.5; 56% of explained variation). Most of the hatchings were recorded during Day 2, except for at 14 °C where hatchings mostly occurred during Day 3. Hatchings at each temperature were mainly recorded during the same observation period every day. Among the six observation periods, 1 and 2 (19:00–23:00 h and 23:00–3:00 h, respectively) were more relevant, since these included at least 62% of the hatchings by temperature (Fig. 2).

Normal swimming behaviours were equally detected at every temperature. The swimming ratio differed significantly among temperatures (*P* < 0.001), with lower ratios found at higher temperatures (Table 2). *Post-hoc* analysis established five groups of temperatures for the swimming ratio: 10 °C, 14 °C/18 °C, 22 °C, 26 °C and 30 °C (*P* < 0.05). In addition, at higher temperatures fewer oncomiracidia were able to swim for over half of their life (ratio higher than 50%); this reduction was detected up to 30 °C, where none swam for more than 50% of their life.

**Table 2:** Larval longevity and swimming ratio of *Sparicotyle chrysophrii* by environmental factor

Environmental factor	Factor level	N^a^	Survival period (h)	Swimming ratio (%)
			Mean ± SD (Range)	Mean ± SD (Range)
Temperature (± 0.1 °C)	10	44^b^	33.5 ± 28.0 (4–104)^b^	72.6 ± 22.6 (12.5–96.2)^b^
	14	273	20.5 ± 17.7 (0–100)	61.9 ± 24.5 (0–94.7)
	18	280	17.3 ± 13.9 (0–72)	61.4 ± 25.4 (0–93.8)
	22	281	12.9 ± 9.0 (0–52)	53.7 ± 26.3 (0–92.3)
	26	232	9.5 ± 7.1 (0–32)	43.9 ± 24.9 (0–85.7)
	30	20	3.6 ± 2.2 (0–8)	18.8 ± 10.3 (0–25.0)
pH (± 0.1)	7.0	168	7.7 ± 7.0 (0–40)	37.6 ± 22.9 (0–90.0)
	7.9	281	12.9 ± 9.0 (0–52)	53.7 ± 26.3 (0–92.3)
Light regime (light:darkness)	12:12	281	12.9 ± 8.7 (0–52)	54.5 ± 23.5 (0–88. 9)
	0:24	268	12.8 ± 9.3 (0–48)	57.1 ± 23.1 (0–90.0)
	24:0	249	12.4 ± 7.5 (0–40)	52.9 ± 24.7 (0–92.3)
Salinity(ppt)-Temperature (± 0.1 °C)	27−18	134	12.9 ± 8.5 (0–36)	57.7 ± 22.2 (0–88.9)
	36−18	128	15.8 ± 8.7 (4–40)	62.6 ± 20.0 (12.5–90.0)
	37−18	98	31.6 ± 19.1 (4–84)	77.3 ± 21.2 (12.5–95.2)
	38−18	122	26.1 ± 12.4 (4–56)	76.5 ± 16.6 (12.5–92.9)
	47−18	155	13.4 ± 9.5 (0–44)	55.9 ± 28.2 (0–90.9)
	27−22	128	13.3 ± 14.1 (0–76)	48.5 ± 33.3 (0–90.9)
	36−22	112	13.4 ± 6.5 (4–48)	61.0 ± 17.0 (12.5–91.7)
	37−22	123	13.9 ± 7.0 (4–32)	61.6 ± 20.0 (25.0–87.5)
	38−22	103	13.7 ± 10.6 (4–44)	51.9 ± 25.5 (25.0–90.9)
	47−22	97	15.6 ± 13.8 (0–44)	53.3 ± 31.5 (0–90.9)

^a^N, number of emerged oncomiracidia used to calculate the survival period and swimming ratio

^b^Includes replicates with emerged eggs (R = 3)

**Fig. 3:**
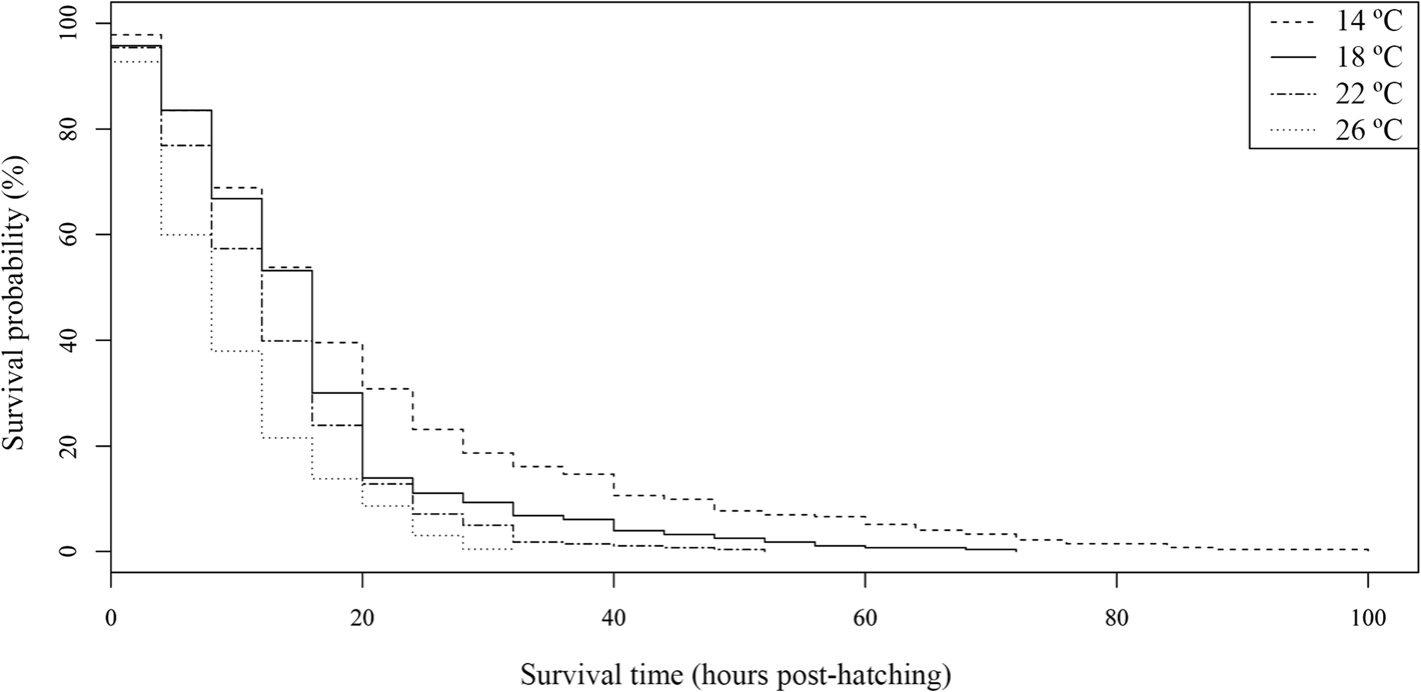
Kaplan-Meier survival curves for *Sparicotyle chrysophrii* oncomiracidia incubated at four temperatures

The oncomiracidial survival period was also affected by water temperatures. Mean and maximum periods were shorter at higher temperatures (Table 2). However, the minimum survival period was 0 h at all temperatures and replicates (Additional file 2: Table S2). Significant differences were detected between the Kaplan-Meier survival curves by temperature (*P* < 0.05). These curves revealed that increasing temperatures had a negative effect on survival (Fig. 3). A similar effect was found in the Cox model for larval survival by temperature, since mortality risk was significantly higher at all the temperatures compared to 10 °C (Table 3).

**Table 3:** Result of the Cox proportional hazards model for the survival period at each temperature

Model term (°C)	Estimate	SE	HR	*P*-value
14	0.5686	0.1676	1.766	<0.001
18	0.7512	0.1698	2.120	<0.001
22	1.0628	0.1720	2.895	<0.001
26	1.3994	0.1752	4.053	<0.001
30	2.3716	0.2807	10.715	<0.001

*Abbreviations*: HR, hazard ratio values for each level of the factor evaluated compared to the reference level (10 °C); SE, standard error

### Experiment 2: Assessment of the pH effects

The influence of pH on the embryonic development of *S. chrysophrii* was slight, and its duration was similar for both, pH 7.0 and 7.9. Hence, time to detection of the first eye-spots was similar at both pH levels, differing by less than 24 h (96 h at pH 7.9 and 120 h at pH 7.0).

**Fig. 4:**
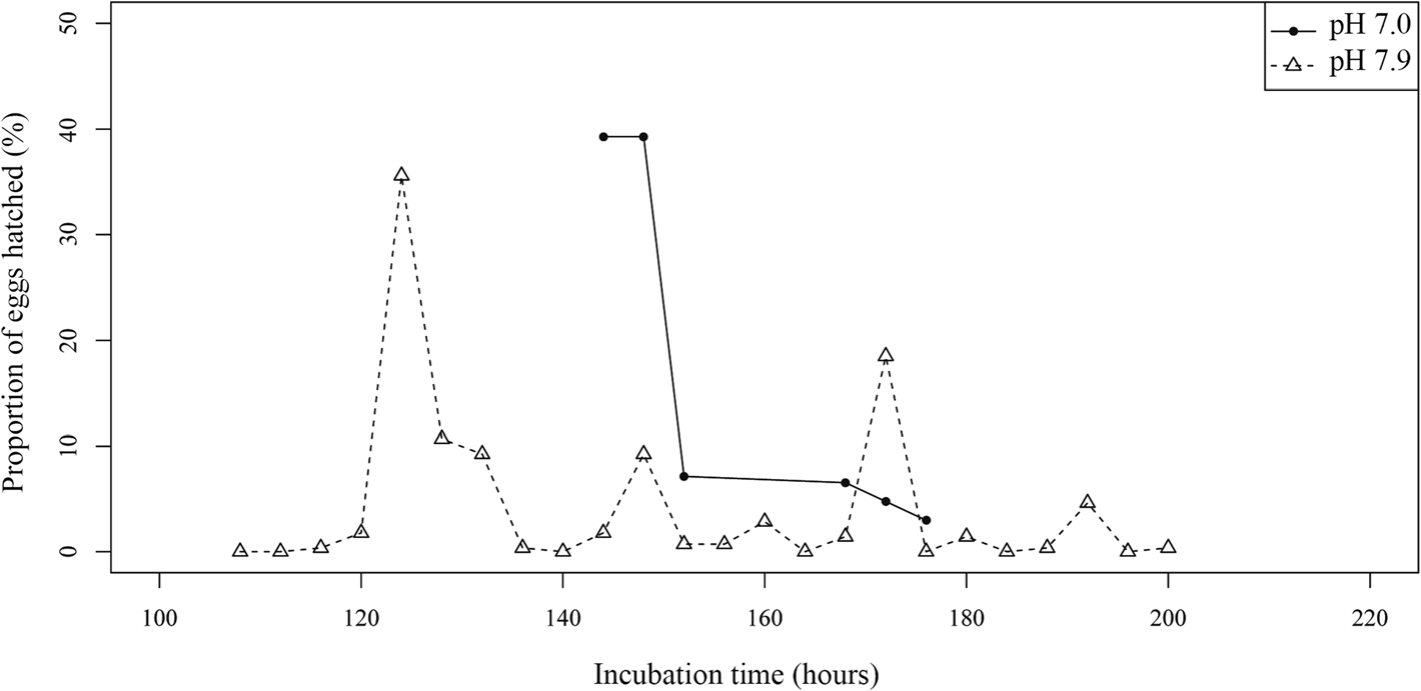
Proportion of hatched *Sparicotyle chrysophrii* eggs at each observation moment, incubated at two pH levels

Slight differences were generally found on incubation periods between pH levels and wells (Table 1 and Fig. 4), except for one replicate at pH 7.9 in which this period was markedly shorter (Additional file 3: Table S3). However, these differences on incubation periods were statistically significant between both pH levels (*P* < 0.001) as well as among replicates incubated at each pH (*P* < 0.001).

The hatching period was shorter at pH 7.0 than at pH 7.9 and this trend was also consistent for the replicates within pH levels (Table 1 and Additional file 3: Table S3). Hatching peaks were mainly detected during the first third of the hatching period for both pH levels (Fig. 4), which corresponded to the first 10 h at pH 7.0 and 25 h at pH 7.9. Hatching success was significantly affected by pH (*P* < 0.001) since fewer eggs hatched at pH 7.0 than at pH 7.9.

**Fig. 5:**
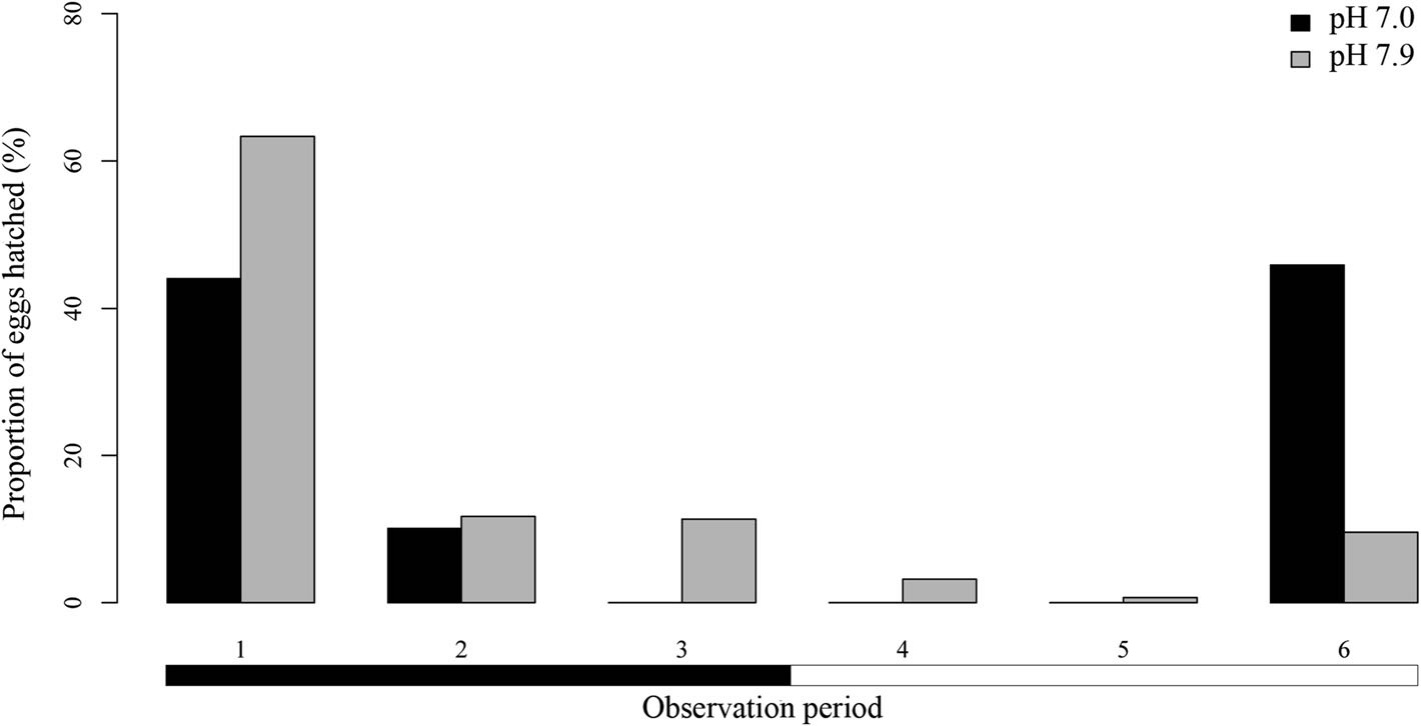
Proportion of hatched *Sparicotyle chrysophrii* eggs for each observation period, incubated at two pH levels. Horizontal bars below the chart indicate darkness (black) and light (white)

Focusing on hatching distribution, more than the 54% of the hatchings were recorded during the dark periods at both pH levels (Fig. 5). However, the best model, which combined number of day (*P* < 0.001) and observation period (*P* < 0.001), was enough to explain 66% of the variation (AIC = 212.5). Most of the hatchings were recorded during Day 2 for both pH levels and daily at the same observation periods, the period 1 (19:00–23:00 h) and period 2 (23:00–3:00 h), which included more than 54% of the hatchings (Fig. 5).

Normal swimming behaviours were observed at both pH levels. Moreover, significant differences were found on swimming ratios between pH levels (*P* < 0.05), with lower ratios recorded at pH 7.0 than at pH 7.9 (Table 2). The number of oncomiracidia swimming for more than half of their life (ratio higher than 50%) was also lower at pH 7.0 than at pH 7.9.

**Fig. 6:**
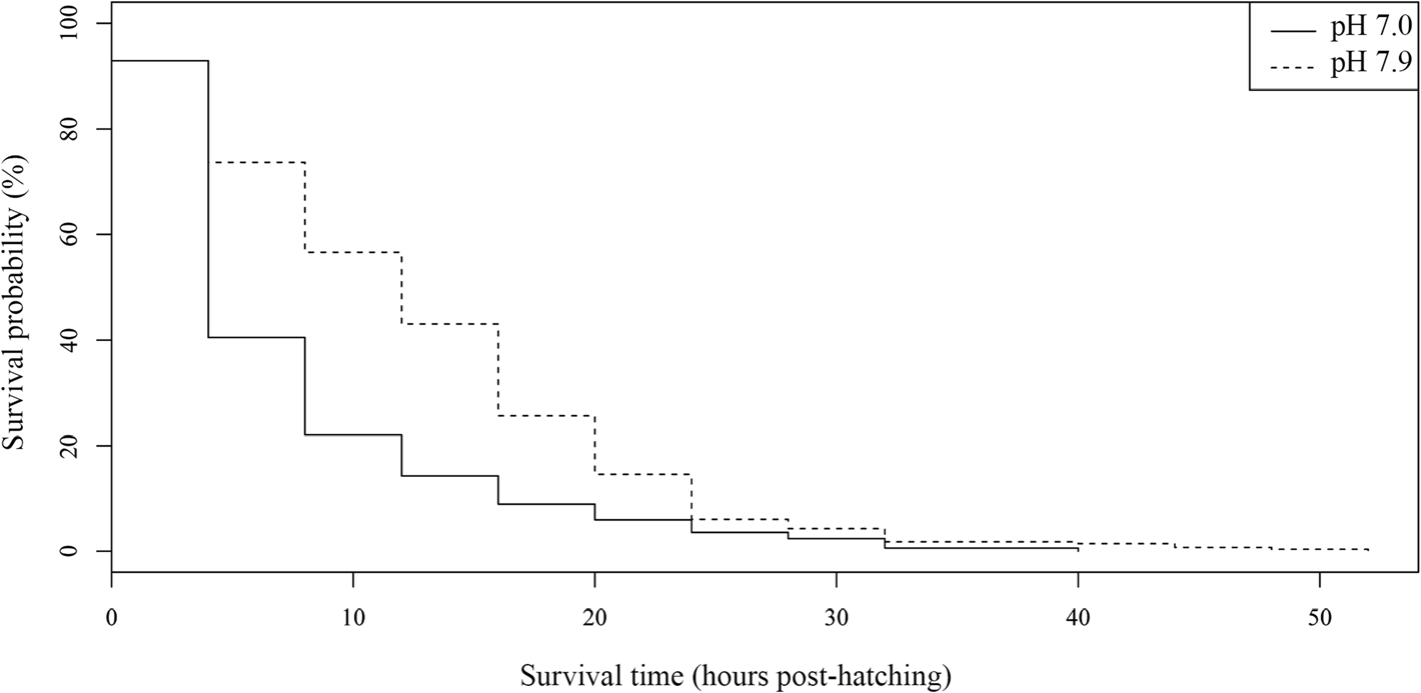
Kaplan-Meier survival curves for *Sparicotyle chrysophrii* oncomiracidia incubated at two pH levels

Sea water pH also influenced the oncomiracidia survival period (Table 2, Additional file 4: Table S4). The mean as well as the maximum survival periods were shorter at pH 7.0 than at pH 7.9, which was also registered for the individual replicates, while the minimum incubation period was 0 h for both pH levels. Kaplan-Meier survival curves were significantly different between pH levels (*P* < 0.001) (Fig. 6). Similarly, the Cox model revealed that mortality risk was significantly higher at pH 7.0 than at pH 7.9 (Table 4), although it did not fulfil the proportional hazard assumption.

**Table 4:** Result of the Cox proportional hazards model for survival period at each pH level

Model term	Estimate	SE	HR	*P*-value
pH 7.9	-0.5160	0.1001	0.5969	<0.001

*Abbreviations*: HR, hazard ratio values for each level of the factor evaluated compared to the reference level (pH 7.0); SE, standard error

### Experiment 3: Assessment of the photoperiod effects

Light regime had only a minor influence on embryonic development since similar developmental times were recorded for each regime (LD 12:12, 0:24 and 24:0). Indeed, eye-spots were detected simultaneously in the three light regimes after 96 h.

**Fig. 7:**
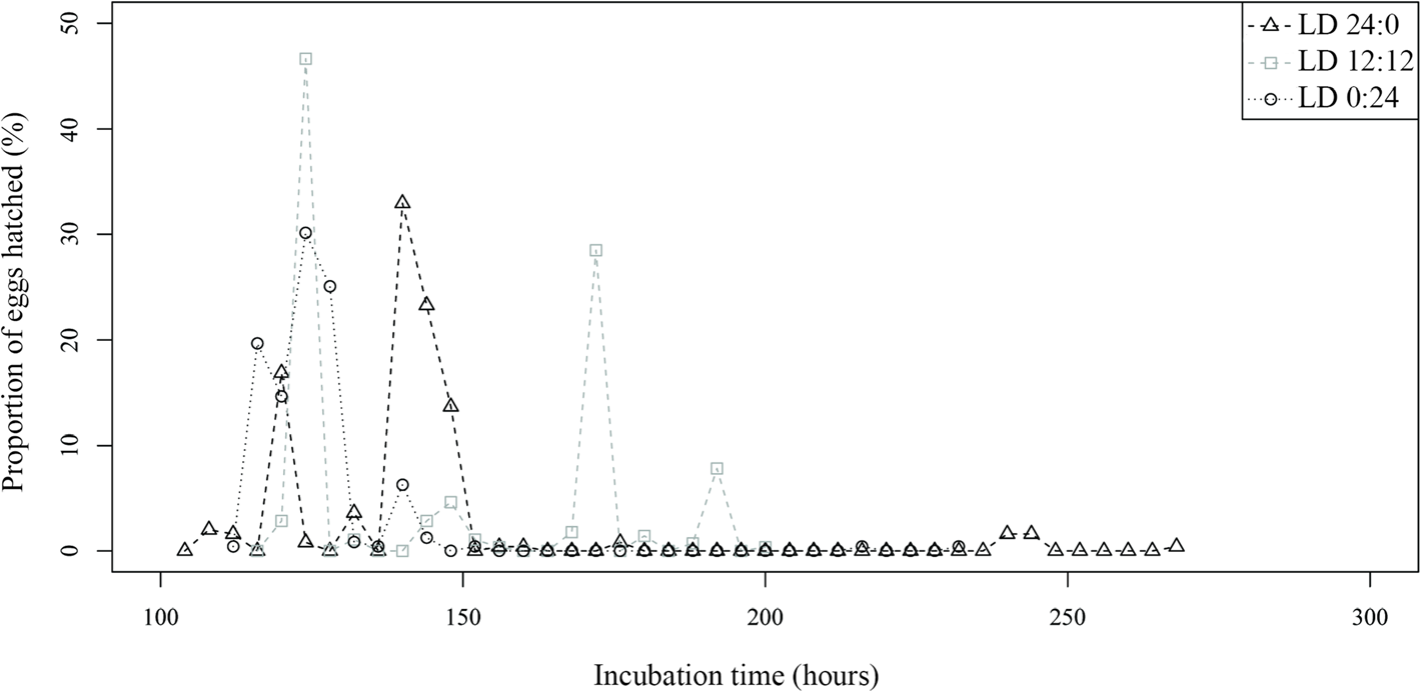
Proportion of hatched *Sparicotyle chrysophrii* eggs at each observation moment, incubated at three light regimes

Incubation period was similar among the light regimes (Table 1 and Fig. 7). However, significant differences were found among the three conditions (*P* < 0.001). *Post-hoc* analysis established two groups of photoperiods for the incubation period: LD 12:12/ LD 0:24 and LD 24:0 (*P* < 0.001). Large and significant variability in the incubation period was found among replicates (*P* < 0.05) (Additional file 5: Table S5).

Hatching periods were longer under constant light (LD 24:0) and dark (LD 0:24) than under alternating light conditions (LD 12:12); however, replicates within each light regime were very different among them (Table 1 and Additional file 5: Table S5). In addition, egg hatching was advanced by approximately 24 h in the constantly dark condition with respect to the other light regimes. Hatching peaks were mostly recorded during the first third of the hatching period for all replicates in each light regime (Additional file 5: Table S5). Similar hatching success was recorded under constant and alternating light conditions (Table 1 and Additional file 5: Table S5). Accordingly, no significant differences in hatching success were detected among the three light regimes.

**Fig. 8:**
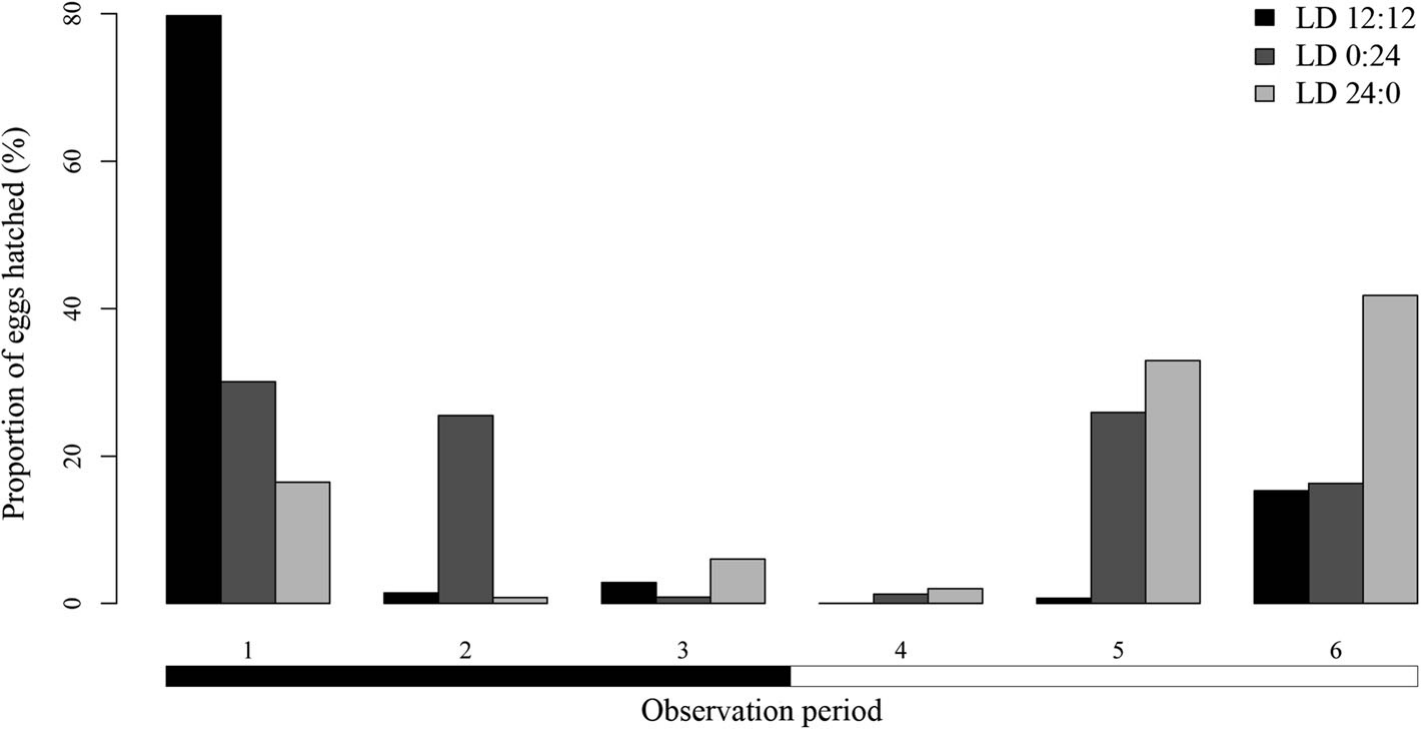
Proportion of hatched *Sparicotyle chrysophrii* eggs for each observation period, incubated at three photoperiods. Horizontal bars below the chart indicate darkness (black) and light (white)

Hatching distribution among observation periods of 12 h, corresponding to light and dark periods in LD 12:12, also differed among light regimes. Henceforth, for LD 0:24 and LD 24:0, these 12 h periods will be referred to as light and dark periods. More than 56.4% of the hatchings occurred during dark periods under LD 12:12 and LD 0:24, although this percentage decreased to 23.3% under LD 24:0 (Fig. 8). Both, number of day (*P* < 0.001) and observation period (*P* < 0.001) had a significant effect on number of hatchings by period in the only model selected: LD 12:12 (AIC = 99.7; 95% of explained variation); LD 0:24 (AIC = 295.0; 70% of explained variation); and LD 24:0 (AIC = 311.8; 65% of explained variation). Hatchings occurred mainly during Day 2 and in the same observation period every 24 h. Regarding the observation periods previously stated, at least 57% of the hatchings were recorded during periods 6 (15:00–19:00 h) and 1 (19:00–23:00 h) regardless of photoperiod, but the highest number of hatchings were detected during the period 1 under LD 12:12 and LD 24:0, and during the period 6 for LD 0:24 (Fig. 8).

Normal swimming behaviours were observed under the three light regimes, and no significant differences in swimming ratio were found for oncomiracidia reared under the three photoperiods (*P* > 0.05). Moreover, a greater number of oncomiracidia swam for more than half of their life under constant darkness (LD 0:24) than in other light regimes.

**Fig. 9:**
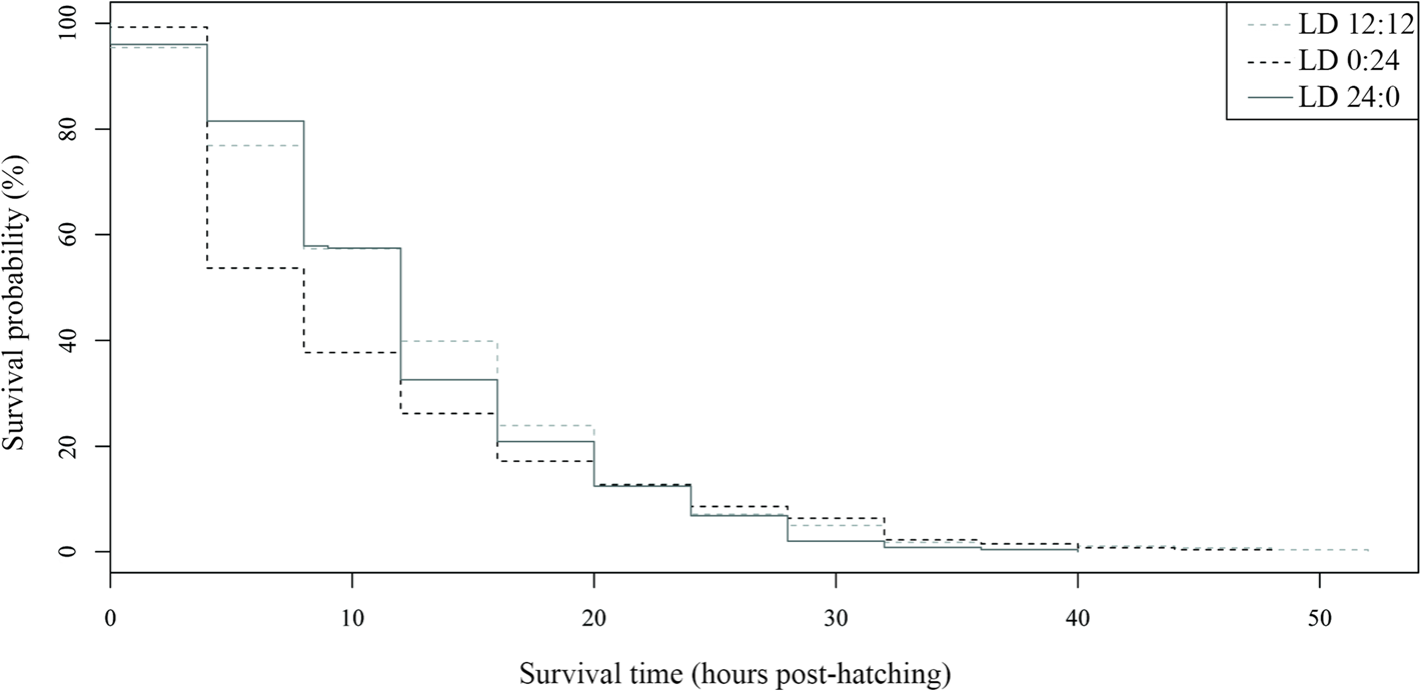
Kaplan-Meier survival curves for *Sparicotyle chrysophrii* oncomiracidia incubated at three photoperiods

Survival periods were similar for oncomiracidia reared under each of the three photoperiods. Mean and minimum survival periods were similar under all light conditions, about 12 and 0 h, respectively, but the maximum survival period was shorter at LD 24:0 than with the other light regimes (Table 2). However, high variability was detected since significant differences were detected among replicates in two of the three light regimes tested (*P* < 0.05) (Additional file 6: Table S6). Significant differences were not found between the Kaplan-Meier survival curves of the three light regimes (*P* > 0.05) (Fig. 9) and the Cox model showed no significant differences in mortality risk among the three light regimes (Table 5).

**Table 5:** Result of the Cox proportional hazards model for survival period within each light regime

Model term	Estimate	SE	HR	*P*-value
LD 0:24	-0.0296	0.0859	0.9708	0.730
LD 24:0	0.0696	0.0874	1.0721	0.426

*Abbreviations*: HR, hazard ratio values for each level of the factor evaluated compared to the reference level (LD 12:12); SE, standard error

### Experiment 4: Assessment of the combined salinity and temperature effects

Embryonic development of *S. chrysophrii* was affected differently by temperature and salinity. All results related to temperature in this experiment agreed with those recorded in experiment 1, thus faster embryonic development was detected at the highest temperature. The only pattern found for the effect of salinity on embryonic development was a slower developmental time at extreme salinities (hypersaline and hyposaline conditions) within each temperature. Eye-spots were firstly detected between 96 and 144 h. The first eye-spots were detected earlier at 22 °C than at 18 °C but for both temperatures the first detection occurred at 36 ppt and the last at 47 ppt.

**Fig. 10:**
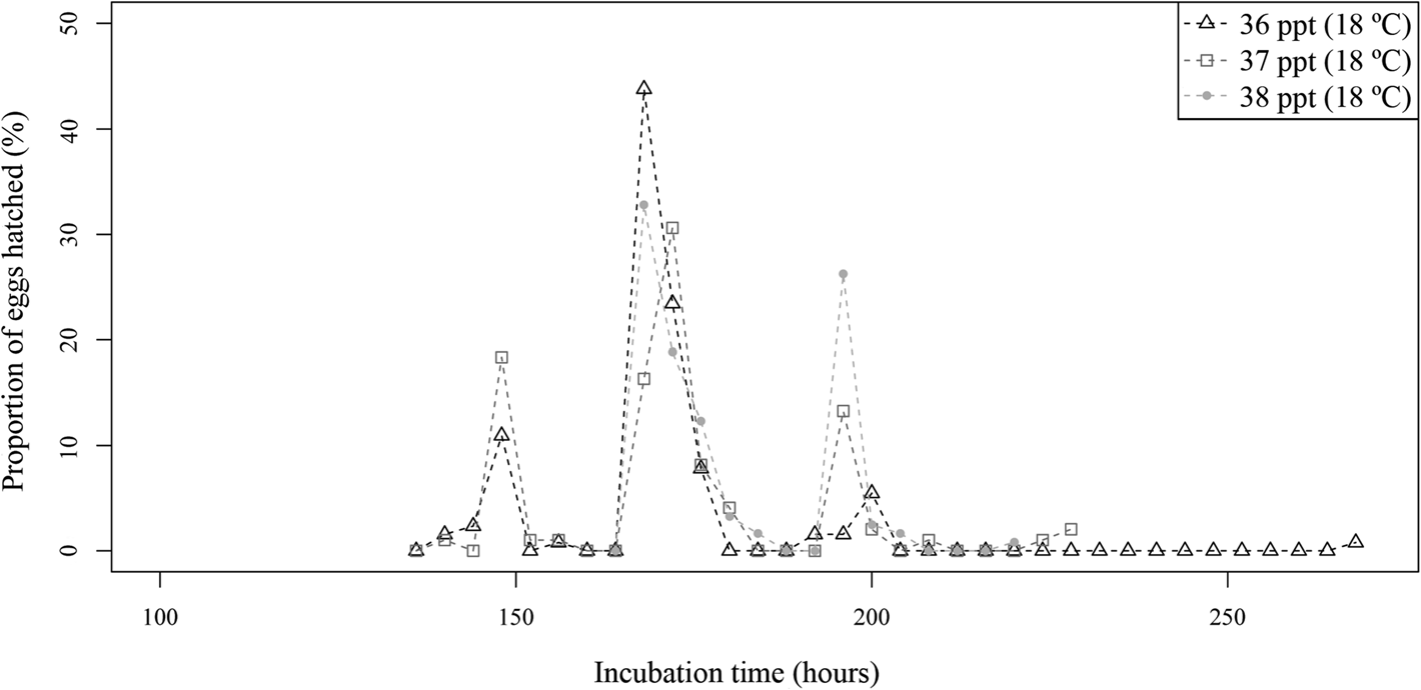
Proportion of hatched *Sparicotyle chrysophrii* eggs at each observation moment, incubated at three salinities at 18 °C

**Fig. 11:**
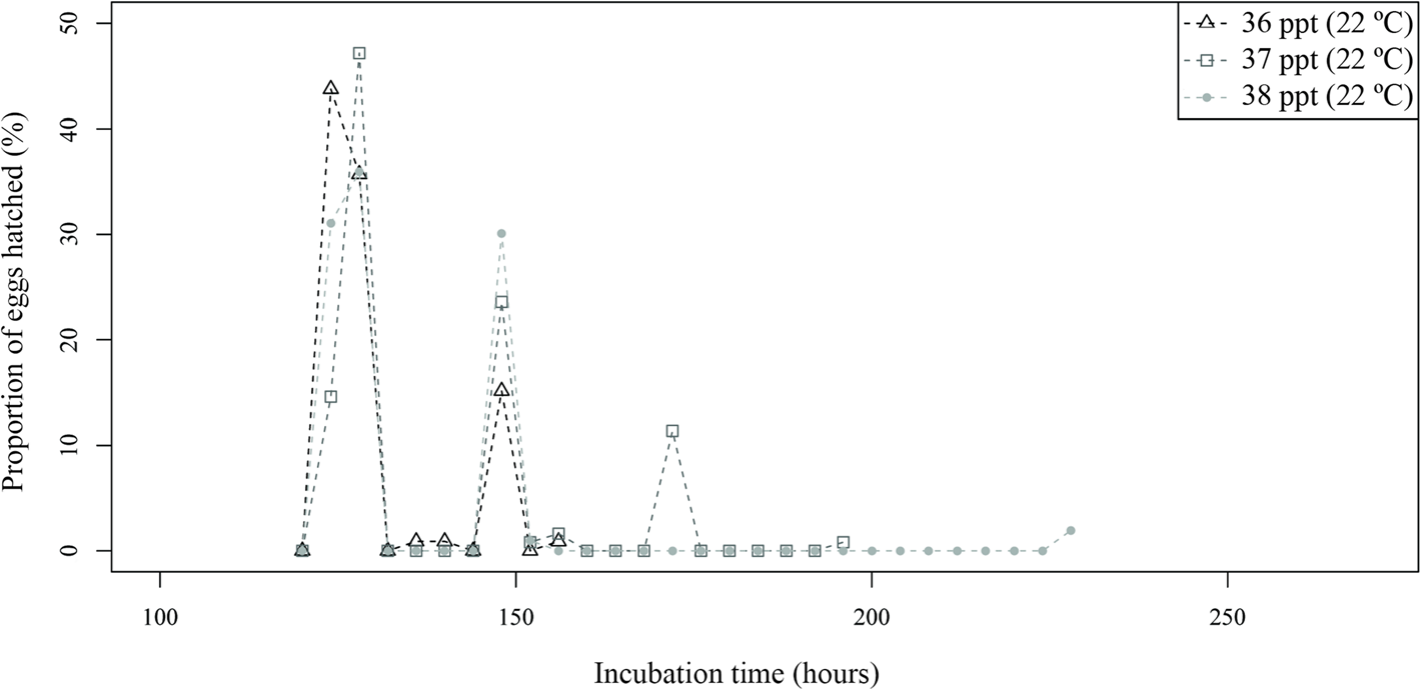
Proportion of hatched *Sparicotyle chrysophrii* eggs at each observation moment, incubated at three salinities at 22 °C

Incubation period was shorter at 22 °C than at 18 °C, as in Experiment 1. This period was also shorter at 36 ppt than at the other common salinities within each temperature (Table 1 and Figs. 10 and 11). Incubation periods at the extreme salinities were longer than at common salinities, with the longest period being detected at 47 ppt for both temperatures (Table 1). Only one out of the four models to explain incubation period was retained. This model (AIC = 9381.9) explained approximately 80% of the variation and included temperature, salinity and the two-way interaction between both although the model without interaction already explained 78%. According to the selected model, the incubation period of the larvae reared at 22 °C was significantly shorter than at 18 °C (*P* < 0.001) and significantly larger at 27 ppt, 38 ppt and 47 ppt than at 36 ppt (*P* < 0.001). Additionally, the interaction term showed that this period was significantly shorter at 27 ppt at 22 °C (*P* < 0.001) and longer at 37 ppt and 47 ppt at 22 °C (*P* < 0.05) than predicted by only the additive effect of both factor levels. Significant differences in the incubation period were also recorded among replicates at each salinity at 22 °C, whereas most salinity replicates at 18 °C were homogeneous (Additional file 7: Table S7).

Hatching periods were also longer at 18 °C than at 22 °C, although a general pattern was not found among salinities within each temperature. At 18 °C, the briefest hatching period was registered at 38 ppt and the longest at 36 ppt. In contrast, at 22 °C, the shortest hatching period occurred at 36 ppt/27 ppt and the longest at 38 ppt. (Table 1 and Additional file 7: Table S7). Hatching peaks were generally detected during the first half of the hatching period (Table 1 and Figs. 10 and 11), but high variability was found among replicates (Additional file 7: Table S7). The total percentage of eggs hatched was similar at both temperatures tested (approximately 75%). However, no pattern was detected for hatching success among the different salinities, since high variability was detected among and within temperatures by salinity as well as by replicates (Table 1 and Additional file 7: Table S7). One of the four models considered for hatching success was retained (AIC = 261.3). Despite this model showing significant effects of temperature, salinity and the two-way interaction between both factors (*P* < 0.05), it barely explained variability in hatching success (less than 32%). According to this model, hatching success was significantly lower at 22 °C than at 18 °C, and at 37 ppt and 47 ppt than at 36 ppt (*P* < 0.05), and the 22 °C and 37 ppt interaction was significantly higher than predicted.

**Fig. 12:**
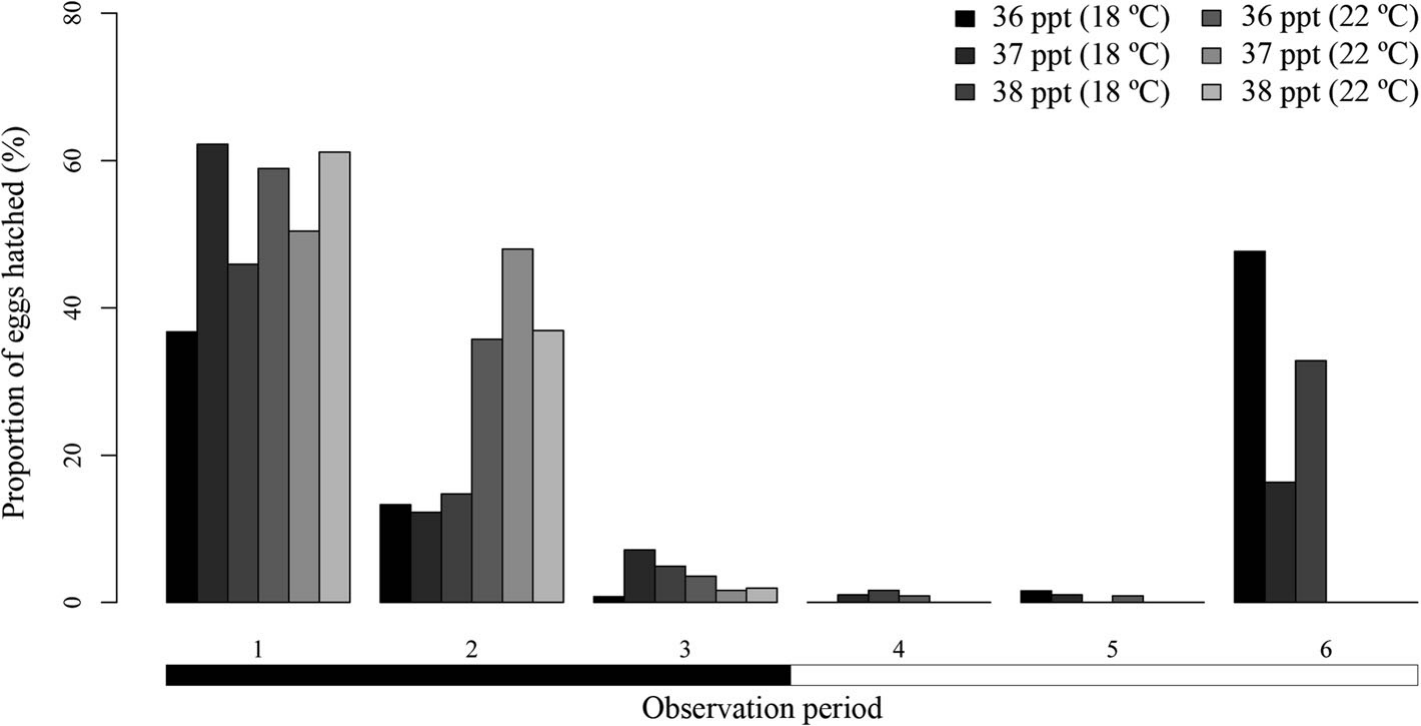
Proportion of hatched *Sparicotyle chrysophrii* eggs for each observation period, incubated at three salinities and two temperatures. Horizontal bars below the chart indicate darkness (black) and light (white)

More than 80% of the hatchings were registered during dark periods for 7 out of 10 temperature and salinity combinations (Fig. 12), while 3 out of 10 showed a hatching success between 50% and 66% (36 ppt and 38 ppt at 18 °C and 27 ppt at 22 °C) (Fig. 12). Number of day (*P* < 0.001) and observation period (*P* < 0.001) had a significant effect on number of hatchings by period. Both explained more than 70% of the variation for all salinities at 18 °C (AICs = 174.1 at 36 ppt, 126.0 at 37 ppt, 69.3 at 27 ppt and 92.5 at 47 ppt), except for 38 ppt (AIC = 220.1 and 54% of explained variation), and at 22 °C (AICs = 86.5 at 36 ppt, 132.1 at 37 ppt, 61.6 at 38 ppt, 111.3 at 27 ppt and 101.5 at 47 ppt). Most of the hatchings were generally registered during Day 2 in the different combinations of temperature and salinity. Hatchings were not uniformly distributed among the six observation periods previously detailed. In general, period 6 (15:00–19:00 h) and period 1 (19:00–23:00 h) accounted for more than 68% of hatchings, regardless of the combination of salinity and temperature tested (Fig. 12). When period 2 was also considered (15:00–19:00/19:00–23:00/23:00–3:00 h), this percentage increased to 92%. However, the significance of these three periods differed among salinities and temperatures with hatchings occurring mainly around light-dark transition (15:00–19:00 and 19:00–23:00 h) for all salinities at 18 °C whilst dark periods (19:00–23:00 and 23:00–3:00 h) were more relevant for salinities at 22 °C (Fig. 12).

Similar to the previous experiments, all oncomiracidia exhibited normal swimming behaviours for every replicate regardless of salinity and temperature. However, significant differences were found in the swimming ratio among the different salinities and temperatures tested. According to the AIC, only one of the four models was retained to assess the swimming ratio (AIC = 15681.0). This model included temperature and salinity as well as the interaction between both factors but explained less than 10% of the variability in the swimming ratio. Based on the model selected, the swimming ratio was significantly higher for 37 ppt and 38 ppt and significantly lower for 27 ppt than at 36 ppt (*P* < 0.001). Likewise, the interaction term showed a significantly lower swimming ratio at 37 ppt and 38 ppt at 22 °C than predicted (*P* < 0.001). These differences were consistent with the data analysis by replicates, although significant variability among them was detected at the common salinities (37 ppt at 18 °C and 36 ppt at 22 °C) as well as at the extreme salinities (27 ppt at both temperatures) (*P* < 0.001). Additionally, the number of oncomiracidia that swam for more than half of their life was higher at 18 °C than at 22 °C as well as at 37 ppt than at other salinities when the data at each temperature were individually analysed.

**Fig. 13:**
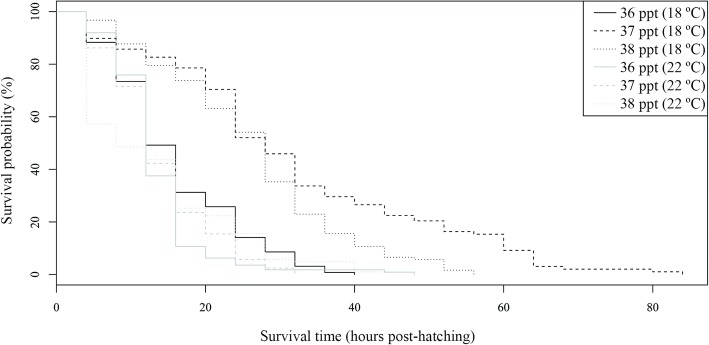
Kaplan-Meier survival curves for *Sparicotyle chrysophrii* oncomiracidia incubated at three salinities and two temperatures

Survival period also differed among salinities and temperatures. In general, longer survival periods were recorded at 18 °C than at 22 °C, but different patterns were found among salinities at each temperature. Whereas mean and maximum survival periods differed among salinities at 18 °C, with higher values being recorded at 37 ppt than at the other salinities, mean and maximum survival periods were similar for the common salinities at 22 °C (Table 2 and Additional file 8: Table S8). Focusing on extreme salinities, differences from common salinities were only found for mean survival period at 27 ppt which was generally lower, but no differences were found for maximum survival period (Table 2 and Additional file 8: Table S8). Differences between common salinities and extreme salinities were also found in minimum survival period, 0 and 4 h, respectively (Table 2 and Additional file 8: Table S8). Kaplan-Meier survival curves were significantly different between temperatures, as well as between salinities and the combination of both factors (*P* < 0.05) (Fig. 13). The only Cox model retained (AIC = 14156.8) revealed that mortality risk was significantly affected by temperature, salinity and the interaction of both factors, but it did not fulfil proportional hazard assumption. Risk of death for the oncomiracidia incubated at 37 ppt and 38 ppt was significantly lower than at 36 ppt. The interaction term showed that this risk was also lower at 27 ppt and 47 ppt at 22 °C (*P =* 0.034; *P =* 0.006) and higher at 37 ppt and 38 ppt at 22 °C (*P* < 0.001; *P =* 0.002) than predicted by the additive effect of these temperature and salinity levels (Table 6).

**Table 6:** Result of the best Cox proportional hazards model for survival period at each salinity within temperature

Best model terms	Estimate	SE	HR	*P*-value	AIC_BEST_
22 °C	0.2341	0.1301	1.2638	0.071	14156.79
37 ppt	-1.0113	0.1434	0.3637	<0.001	
38 ppt	-0.6625	0.1283	0.5155	<0.001	
27 ppt	0.2307	0.1240	1.2595	0.063	
47 ppt	0.1727	0.1295	1.1885	0.182	
22 °C*37 ppt	0.9604	0.1944	2.6127	<0.001	
22 °C*38 ppt	0.5888	0.1868	1.8017	0.002	
22 °C*27 ppt	-0.3832	0.1810	0.6816	0.034	
22 °C*47 ppt	-0.5209	0.1919	0.5940	0.006	

*Abbreviations*: AIC_BEST_, Akaike value of the best model; HR, hazard ratio values for each level of the factor evaluated compared to the reference level [36 ppt (18 °C)]; SE, standard error

## Discussion

To our knowledge, the present study is the most comprehensive analysis of the influence of environmental factors on development, hatching, swimming and survival of the infective stages of monogeneans under consideration of climate change predictions. Moreover, this study constitutes the first *in vitro* research assessing the effects of water pH on the free-living stages of monogeneans and applying survival analysis on them.

In accordance with previous studies on monogeneans [10, 42], temperature was the most significant abiotic factor influencing *S. chrysophrii* biology and modifying every biological parameter evaluated. The most remarkable result of this study is that the free-living stages of *S*. *chrysophrii* are quite sensitive to water temperature, from which increases result in shorter times of occurrence for every biological event. These results are consistent with those reported for other monogenean species [10, 11] and could be related to the effect of temperature on increasing metabolic rates [7, 43]. Subsequently, development of *S*. *chrysophrii* oncomiracidia could be modulated by water temperature from the beginning of their life in order to optimise their adaptation to the changing environmental conditions. This early versatility of free-living stages could also be extended to the parasitic stages (post-larvae and adults) since temperature affects every aspect of the monogeneans’ reproductive and developmental cycles [6, 10, 11, 13–15, 44]. Consequently, it is essential to explore the effect of temperature and other environmental factors on the post-larval development, maturity and fecundity of *S. chrysophrii* to determine the global adaptability of this species to changing environmental conditions.

However, the developmental versatility of *S. chrysophrii* appears to be restrained to a specific temperature range, as illustrated by records of the minimum incubation period. Mean incubation periods of *S. chrysophrii* were generally consistent with those previously reported [34, 35] (but see [45] for data out of current ranges). Likewise, the trend of shorter mean incubation periods at increasing temperatures herein recorded is in agreeance with that reported for other monogeneans [6, 14, 44]. Nevertheless, the minimum incubation period does not follow this tendency and despite its gradual reduction between 14 °C and 22 °C, it did not drop below 5 days at higher temperatures. This minimum period coincided with that of previous studies within the same temperature range (22 °C [35]), and even with the records at lower temperatures (20 °C [34]). Therefore, endogenous components are likely restraining the effect of temperature on the incubation period, i.e. the time needed by oncomiracidia to develop certain larval structures (hooks, eye-spots and cilia) or acquire their functionality.

Thermal conditions also revealed another biological constraint, in this case on larval emergence. Hatching success of *S. chrysophrii* was high in the range of temperatures commonly recorded in western Mediterranean (over 70% from 14 °C to 26 °C, see Table 1), suggesting that it is well-adapted to the conditions in this region. Previous studies on monogeneans found that hatching success was reduced by temperatures that were higher or lower than an optimal range ([6, 10] and references therein). Accordingly, the optimal range of temperatures for the emergence of *S. chrysophrii* larvae (hatching success over 88%) is wide (from 14 °C to 22 °C); however, at extreme temperatures, hatching success is abruptly reduced. The significant low records for hatching success at 10 °C points to a lower thermal margin for the species at this temperature which coincides with that reported on other monogeneans from regions with comparable thermal variations [6, 13, 46]. For higher temperatures, reduction in hatching success of *S. chrysophrii* was slight at 26 °C but higher at 30 °C, with less than 10% of the eggs hatching. The latter suggests that 30 °C could be considered the upper thermal margin for the hatching of the species. However, temperatures above 26 °C are rarely recorded in the western Mediterranean region [47], and thus they should not be considered as a hazard for *S. chrysophrii* hatching. Moreover, in relation to temperature, a strategy of compromise between hatching success and incubation period is also suggested.

The decrease in sea water pH also seems to affect the free-living stages of *S. chrysophrii*, by reducing their biological times and hatching success. Previous studies concerning effects of pH on infective stages of platyhelminths are scarce and did not report consistent trends among species. In general, decreased pH reduces longevity of digenean cercariae by increases of demands to maintain the acid-base balance, although this reduction is variable depending on the species [16, 17, 48]. Focusing on monogeneans, data on the influence of pH is currently based on a few studies on *Pseudodactylogyrus* spp., and no references are available about their infective stages. According to these studies, decreased pH in a sample zone is related to low monogenean infection levels [49, 50] but variations of pH at a small scale do not affect infection levels [51]. Therefore, studies reviewing these results disagree in the estimation of the pH effect on monogeneans [52, 53]. To interpret the results of the current study, the limited sample size due to the low hatching success, and collateral effects of the method employed to reduce pH (such as decreased alkalinity) [54], should also be considered. Moreover, despite hatching success being significantly reduced at pH 7.0, the percentage of larvae that emerged was over 50%. These data suggest that embryonic development in *S. chrysophrii* could tolerate a reduction in pH, at least to one unit below that of the Mediterranean Sea, although further analysis using different analytical methods are required to ascertain this hypothesis.

Hatching of *S. chrysophrii* showed a circadian periodicity regardless of the environmental factor assessed, so other type of factors could be operating. Hatching rhythms have also been reported for other monogenean species (*Entobdella soleae*, *Rajonchocotyle emarginata*, *Benedenia lutjani* and *B. rohdei*) by Kearn [55], Whitthington & Kearn [56] and Ernst & Whittington [57], who postulated its relationship with endogenous stimuli operating at cellular or molecular levels. Most hatchings of *S. chrysophrii* occurred during the period 1 for every replicate and experiment submitted to the dark condition (LD 12:12, LD 0:24). Nevertheless, in replicates without darkness (LD 24:0), the period 6 included most of the hatchings. Thus, darkness seems to influence larval emergence of this species beyond hatching rhythms, as it synchronised most of the hatchings with the night (dark periods), particularly during the period 1, and to a lesser degree with Day 2. This hypothesis is consistent with previous studies on *Neoentobdella diadema* and *Plectanocotyle gurnardi*, which highlighted the role of light as an environmental cue for hatching [58, 59]. Therefore, regulation of larval emergence by light conditions would occur before egg hatching, as suggested by Kearn [10], who proposed that the translucent egg shell of monogeneans would allow larvae to detect light before emergence and synchronise hatching. For *S. chrysophrii*, detection of light cues could be related to development of larval eye-spots, from 24 to 48 hours before hatching.

Embryonic and larval development of *S. chrysophrii* are also influenced by salinity, which affects the incubation and survival periods as well as the swimming ratio. However, results were sometimes conflicting between temperatures, and high variability was found among individuals and replicates, thus no trend was distinguished. Taking into account these results, the influence of salinity on free-living stages of *S. chrysophrii* could be considered negligible. The low influence of salinity variations around the mean value of a particular region generally agreed with previous studies on other marine monogeneans [15, 20–23, 60]. However, these studies recorded notable effects of wide salinity variations, especially at hypersaline conditions when hyper- and hyposaline deviations are comparable. Infective stages of *S. chrysophrii* were not noticeably affected by any of the extreme salinities (47 ppt and 27 ppt) and thus this species likely has a high degree of tolerance to salinity variations.

High individual variability was detected for some parameters among levels and replicates for each factor assessed (temperature, pH, light and salinity). For example, variability between specimens found at a specific temperature in Experiment 1 (*n* = 300) included the entire range of individual variability for each combination of temperature-salinity in Experiment 4 (*n* = 150), even when significant differences were detected. These differences could be associated with sample size, thus a higher number of specimens would be needed to determine the real influence of salinity on this species. However, most studies analysing the influence of abiotic factors on monogeneans have been developed using few specimens, thus results should be interpreted with caution. Future studies should consider more than one replicate for each environmental condition and a sample size wide enough to detect effects beyond individual variability.

By integrating the results for *S. chrysophrii* and related information on the behaviour of gilthead sea bream, two main strategies for larval development and hatching, which would allow the parasite to synchronise with the host and improve transmission, can be highlighted. Wild juvenile gilthead sea breams exhibit different distribution patterns depending on seasonality of temperatures [27, 61]. In the spring, fish migrate to shallow and warm water areas where they remain grouped until autumn. Per the thermal effects described in this study, this period would be coincident with fast embryonic development and early hatching of *S. chrysophrii*. This strategy would favour parasite transmission, since the host encounter is facilitated as the fish are grouped. In autumn, the juvenile fish avoid cold temperatures and return to the open sea where they are more dispersed. Under these conditions, the longer embryonic development, hatching period and survival of *S*. *chrysophrii* would be more appropriate to increase the chances for the infective stages to find dispersed hosts. This strategy would be also suitable to infect adult gilthead sea breams, since they live mostly in deeper areas of the open sea, at temperatures between 16–20 °C [47], and remain isolated or slightly aggregated throughout the year, except for autumn when they group for reproductive purposes [27], and the host-finding probability for the parasite would increase.

Similarly, photoperiod has also an influence on *S. chrysophrii* hatching which would also increase host encounter probability in accordance with *S. aurata* behaviour, as suggested by Repullés-Albelda et al. [34]. Larval emergence would preferably occur during dark periods when the host rests [62] and is probably more vulnerable to infection ([10, 12] and references therein). Moreover, light conditions could also coordinate larval emergence with dark periods, which would also reduce the exposure to potential oncomiracidia predators [10, 56, 57].

Parasite-host coordination strategies would be mostly functional with wild fish, whose activity and distribution can be fitted to each environmental condition. In aquaculture, overcrowding increases the probability of host encounter regardless of the season, hence parasite-host synchronisation is not that critical for the parasite to succeed. However, infection success in monogeneans entails not only host finding but also host invasion and settlement [8]; in these processes, fish health condition, which is altered by environmental variations and handle stressors [63–65], would play a main role. Studies developed with cultured gilthead sea breams show that their condition can be notably altered by variations of water temperature [66, 67]. At cold temperatures in particular, the immune system of the gilthead sea bream is compromised [66] while increasing temperatures may promote some immune components [67]. Likewise, development and hatching periods of *S. chrysophrii* are long at cold temperatures but become shorter as water temperature increases. These variations in host-parasite conditions could explain the seasonal infection levels reported for *S. chrysophrii* in gilthead sea bream fish farms, where prevalence and intensity are high in spring but remain commonly low, with sporadic peaks (see [68]), during winter months [24, 29, 30]. Therefore, to the extent that environment affects parasite biology and fish condition, different infection levels will be recorded in cultures. Moreover, long-term follow-up of this host-parasite system is required for predicting accurately the seasonal infection dynamics in each region.

Given the influence of temperature on *S. chrysophrii* transmission, knowledge on the effect of water temperature on development of *S*. *chrysophrii* oncomiracidia can help to design therapeutic and prophylactic measures adapted to specific climatic conditions, as well as to reduce the number of treatments in culture facilities from the Mediterranean Sea. Moreover, the nocturnal hatching habits of *S. chrysophrii* should also be considered. Previous studies warned about egg resistance to treatments [32], consequently two treatment administrations are recommended to avoid *S. chrysophrii* for long periods [33, 34]. The first treatment would remove larval, juvenile and adult monogeneans, and the second, in which schedule is crucial, would kill worms emerging from resistant eggs before they grow. In accordance with Repullés-Albelda et al. [34], the period between treatments should be established as the estimated time for all eggs to hatch after treatment, which is calculated by adding the maximum incubation and survival periods. As shown in the present study, these periods change depending on temperature, thus highlighting the importance of this factor to adjust the periods between treatments. According to data reported in this study, the time elapsed between the primary and secondary treatments should be as follows: 10 days (8 of incubation period and 2 of survival period) at 26 °C; 12 days (9 + 3) at 22 °C; 14 days (11 + 3) at 18 °C: and 19 days (14 + 5) at 14 °C. However, since Day 2 or 3 were the most important for hatching, treatment after these periods would be enough to notably reduce reinfection. Since *in vitro* studies only simulate the conditions *in vivo* [14], advice herein reported should be considered as an illustrative aid for treatment design. To improve effectiveness, treatment calendars should be adapted to each aquaculture facility, thus allowing a better adjustment to conditions *in situ* (infrastructure, local currents, depth, country legislation, etc.).

Forecasting the effects of climate change on parasitic infections is problematic and, as a global process, analyses on synergic effects between environmental variables are required [69–71]. However, studies evaluating the influence of specific environmental factors on monogenean biology can be helpful to understand short-term changes in infections. Among all abiotic factors affected by climate change, variations in water temperature, pH and salinity have been reported to modify host-parasite relationships in aquatic environments [69–71]; consequently, the expected alteration of these factors in the western Mediterranean region could modify the *S*. *aurata-S. chrysophrii* infection dynamics. A temperature increase of up to 2.5 °C is predicted by 2100 for the western Mediterranean region [9], which falls within the thermal tolerance range reported for *S*. *aurata* [27] and *S*. *chrysophrii* (present study). To our knowledge, this rise would benefit gilthead sea bream by enhancing its immune system [67] as well as *S. chrysophrii* by accelerating its larval development, as shown in this study. Nevertheless, the constraints recorded for the developmental versatility of *S*. *chrysophrii* based on temperatures should also be kept in mind. *Sparus aurata*, as well as *S. chrysophrii*, appear to have a similar tolerance to high temperatures, with an upper thermal limit of approximately 33 °C and 30 °C, respectively ([27]; present study). Focusing on water acidification, an annual decrease of 0.004 pH units has been estimated for the western Mediterranean, reaching a pH of approximately 7.4 in 2100 [37]. A slight influence of decreases of water pH has been reported on gilthead sea bream [72] and *S. chrysophrii* (present study), suggesting that both organisms would tolerate pH variations predicted to date. However, synergic effects of pH reduction with other factors should be explored for *S. chrysophrii*, since deleterious effects have been reported for gilthead sea bream in acidified sea water combined with temperature [72]. In relation to salinity, an increment of up to 0.5 ppt has been predicted for the next 100 years in the Mediterranean Sea [9]. Therefore, the wide salinity tolerance of gilthead sea bream (from 5 to 45 ppt [27]) makes unlikely that these species will be affected by the salinity increase projected. Despite of the fact that evolutionary potential and adaptability are predicted to be greater for parasites than for hosts [73], acclimation processes are likely to succeed for both organisms as changes are predicted to occur gradually enough [74]. Nevertheless, conditions in aquaculture facilities or in certain local microclimates might alter the stability of the relationship between gilthead sea bream and *S. chrysophrii* with unknown consequences. Therefore, the magnitude and gradualness of abiotic changes, as well as the conditions in specific environments, will determine the progression of this host-parasite relationship under climate changes.

## Conclusions

The findings of this study revealed a high versatility of infective stages of *S. chrysophrii* to variations on abiotic factors. Temperature induced the most remarkable effects on eggs and larvae of this monogenean species since it modifies all biological parameters analysed, whereas the photoperiod mainly affects larval emergence. By contrast the influence of salinity and slight variations of pH was minor. The environmental influence on *S. chrysophrii* seems to play a relevant role on host-parasite coordination and transmission and thus, it should be considered for designing infection management strategies in gilthead sea bream cultures. Despite its environmental susceptibility, *S. chrysophrii* exhibited a high tolerance to the environmental variations predicted under climate change context suggesting a low influence of this climatic process on this monogenean. Previously reported data also suggest a high tolerance of gilthead sea bream to these variations but further integrated studies are required to test the stability of the whole parasite-host system.

## Additional files

Additional file 1: **Table S1.** Parameters of embryonic development of *S. chrysophrii* by replicate at each temperature. (DOCX 15 kb)

Additional file 2: **Table S2.** Larval longevity and behaviour of *S. chrysophrii* by replicate at each temperature. (DOCX 15 kb)

Additional file 3: **Table S3.** Parameters of embryonic development of *S. chrysophrii* by replicate at each pH level. (DOC 37 kb)

Additional file 4: **Table S4.** Larval longevity and behaviour of *S. chrysophrii* by replicate at each pH level. (DOCX 13 kb)

Additional file 5: **Table S5.** Parameters of embryonic development of *S. chrysophrii* by replicate at each light regime. (DOC 36 kb)

Additional file 6: **Table S6.** Larval longevity and behaviour of *S. chrysophrii* by replicate at each light regime. (DOCX 13 kb)

Additional file 7: **Table S7.** Parameters of embryonic development of *S. chrysophrii* by replicate at different salinities and temperatures. (DOC 45 kb)

Additional file 8: **Table S8.** Larval longevity and behaviour of *S. chrysophrii* by replicate at different salinities and temperatures. (DOCX 15 kb)

## Abbreviations

AIC: Akaike information criterion
HR: Hazard ratio
SE: Standard error
SD: Standard deviation

## References

[1] Chernin J (2000). Parasitology.

[2] Poulin R (1996). The evolution of life history strategies in parasitic animals. Adv Parasitol.

[3] Jackson J, Tinsley R (1998). Effects of temperature on oviposition rate in *Protopolystoma xenopodis* (Monogenea: Polystomatidae). Int J Parasitol.

[4] Wiegertjes GF, Flik G (2004). Host-Parasite Interactions.

[5] Koskivaara M (1992). Environmental factors affecting monogeneans parasitic on freshwater fishes. Parasitol Today.

[6] Gannicott A, Tinsley R (1998). Environmental effects on transmission of *Discocotyle sagittata* (Monogenea): egg production and development. Parasitology.

[7] Gannicott A, Tinsley R (1998). Larval survival characteristics and behaviour of the gill monogenean *Discocotyle sagittata*. Parasitology.

[8] Bush AO, Fernández JC, Esch GW, Seed R (2001). Parasitism: The Diversity and Ecology of Animal Parasites.

[9] Otero M, Garrabou J, Vargas M (2013). Mediterranean Marine Protected Areas and Climate Change: A Guide to Regional Monitoring and Adaptation Opportunities.

[10] Kearn GC (1986). The eggs of monogeneans. Adv Parasitol.

[11] Whittington ID, Chisholm LA, Rohde K (2000). The larvae of Monogenea (Platyhelminthes). Adv Parasitol.

[12] Whittington ID, Kearn GC (2011). Hatching strategies in monogenean (Platyhelminth) parasites that facilitate host infection. Integr Comp Biol.

[13] Jackson J, Tinsley R, Du Preez L (2001). Differentiation of two locally sympatric *Protopolystoma* (Monogenea: Polystomatidae) species by temperature-dependent larval development and survival. Int J Parasitol.

[14] Tubbs L, Poortenaar C, Sewell M, Diggles B (2005). Effects of temperature on fecundity *in vitro*, egg hatching and reproductive development of *Benedenia seriolae* and *Zeuxapta seriolae* (Monogenea) parasitic on yellowtail kingfish *Seriola lalandi*. Int J Parasitol.

[15] Brazenor AK, Hutson KS (2015). Effects of temperature and salinity on the life cycle of *Neobenedenia* sp. (Monogenea: Capsalidae) infecting farmed barramundi (*Lates calcarifer*). Parasitol Res.

[16] MacLeod C, Poulin R (2015). Differential tolerances to ocean acidification by parasites that share the same host. Int J Parasitol.

[17] Guilloteau P, Poulin R, MacLeod CD (2016). Impacts of ocean acidification on multiplication and caste organisation of parasitic trematodes in their gastropod host. Mar Biol.

[18] Kearn G (1980). Light and gravity responses of the oncomiracidium of *Entobdella soleae* and their role in host location. Parasitology.

[19] Chisholm L, Whittington I (2000). Egg hatching in 3 species of monocotylid monogenean parasites from the shovelnose ray *Rhinobatos typus* at Heron Island, Australia. Parasitology.

[20] Umeda N, Hirazawa N (2004). Response of the monogenean *Neobenedenia girellae* to low salinities. Fish Pathol.

[21] Ernst I, Whittington ID, Corneillie S, Talbot C (2005). Effects of temperature, salinity, desiccation and chemical treatments on egg embryonation and hatching success of *Benedenia seriolae* (Monogenea: Capsalidae), a parasite of farmed *Seriola* spp. J Fish Dis.

[22] Chen HG, Chen HY, Wang CS, Chen SN, Shih HH (2010). Effects of various treatments on egg hatching of *Dendromonocotyle pipinna* (Monogenea: Monocotylidae) infecting the blotched fantail ray, *Taeniurops meyeni*, in Taiwan. Vet Parasitol.

[23] Grano-Maldonado MI, Aguirre-Villaseñor H, Betancourt-Lozano M, Fajer-Ávila EJ (2015). *In vitro* effect of low salinity on egg hatching and larval survival of *Heterobothrium ecuadori* (Monogenea) infecting bullseye puffer fish *Sphoeroides annulatus*. Aquacult Res.

[24] Faisal M, Imam E, Perkins FO, Cheng TC (1990). *Microcotyle chrysophrii* (Monogenea, Polyopisthocotylea), a pathogen for cultured and wild gilthead seabream, *Sparus aurata*. Pathology in Marine Science.

[25] Sanz F (1992). Mortality of cultured seabream (*Sparus aurata*) caused by an infection with a trematode of the genus *Microcotyle*. Bull Eur Assoc Fish Pathol.

[26] Athanassopoulou F, Ragias V, Vagianou S, Di Cave D, Rigos G, Papathanasiou G (2005). Report of *Sparicotyle* (*Microcotyle*) *chrysophrii* Van Beneden and Hesse 1863, *Atrispinum seminalis* Euzet and Maillard 1973 and *Polylabris tubicirrus* Paperna and Kohn 1964 (Monogenea) on captive sea bream (*Sparus aurata*) and sharp snout sea bream (*Diplodus puntazzo*) in coastal Greece and Italy. Bull Eur Assoc Fish Pathol.

[27] Ortega A (2008). Cultivo de la Dorada (*Sparus aurata*).

[28] APROMAR, Asociación Empresarial de Productores de Cultivos Marinos. La acuicultura marina de peces en España. 2016. http://www.apromar.es/content/informes-anuales. Accessed 17 Jan 2017.

[29] Reversat J, Silan P, Maillard C (1992). Structure of monogenean populations, ectoparasites of the gilthead sea bream *Sparus aurata*. Mar Biol.

[30] Sitjà-Bobadilla A, Redondo MJ, Alvarez-Pellitero P (2010). Occurrence of *Sparicotyle chrysophrii* (Monogenea: Polyopisthocotylea) in gilthead sea bream (*Sparus aurata* L.) from different mariculture systems in Spain. Aquacult Res.

[31] Mahmoud NE, Mahmoud A, Fahmy M. Parasitological and comparative pathological studies on monogenean infestation of cultured sea bream (*Sparus aurata*, Sparidae) in Egypt. Oceanography. 2014;2:129.

[32] Noga EJ (2010). Fish Disease: Diagnosis and Treatment.

[33] Repullés-Albelda A, Raga JA, Montero FE (2011). Post-larval development of the microcotylid monogenean *Sparicotyle chrysophrii* (Van Beneden and Hesse, 1863): comparison with species of Microcotylidae and Heteraxinidae. Parasitol Int.

[34] Repullés-Albelda A, Holzer AS, Raga JA, Montero FE (2012). Oncomiracidial development, survival and swimming behaviour of the monogenean *Sparicotyle chrysophrii* (Van Beneden and Hesse, 1863). Aquaculture.

[35] Euzet L, Noisy D (1979). *Microcotyle chrysophrii* (Van Beneden and Hesse, 1863) (Monogenea, Microcotylidae), a parasite of the teleostean *Sparus aurata*. Morpho-anatomical data of the adult and of the oncomiracidium. Vie Milieu Paris (France).

[36] Oceanographic database. Puertos del Estado. 2014. http://www.puertos.es/es-es/oceanografia/Paginas/portus.aspx. Accessed 21 May 2014.

[37] Flecha S, Pérez FF, García-Lafuente J, Sammartino S, Ríos AF, Huertas IE (2015). Trends of pH decrease in the Mediterranean Sea through high frequency observational data: indication of ocean acidification in the basin. Sci Rep.

[38] Burnham K, Anderson D (2002). Model Selection and Multimodel Inference: A Practical Information-theoretic Approach.

[39] Dinno A. Dunn’s test of multiple comparisons using rank sums. R package version 1.2.0. 2014. http://CRAN.R-project.org/package=dunn.test. Accessed 16 April 2016.

[40] Therneau T. A package for survival analysis in S. R package version 2.38. 2015. http://CRAN.R-project.org/package=survival. Accessed 28 June 2018

[41] R Development Core Team (2014). R: A language and environment for statistical computing. Version 3.1.2.

[42] Chubb JC (1977). Seasonal occurrence of helminths in freshwater fishes Part I. Monogenea. Adv Parasitol.

[43] Tinsley R, Owen RW (1975). Studies on the biology of *Protopolystoma xenopodis* (Monogenoidea): the oncomiracidium and life-cycle. Parasitology.

[44] Cecchini S, Saroglia M, Berni P, Cognetti-Varriale A (1998). Influence of temperature on the life cycle of *Diplectanum aequans* (Monogenea, Diplectanidae), parasitic on sea bass, *Dicentrarchus labrax* (L.). J Fish Dis.

[45] Sitjà-Bobadilla A, de Felipe MC, Alvarez-Pellitero P (2006). *In vivo* and *in vitro* treatments against *Sparicotyle chrysophrii* (Monogenea: Microcotylidae) parasitizing the gills of gilthead sea bream (*Sparus aurata* L.). Aquaculture.

[46] Cecchini S (1994). Influence of temperature on the hatching of eggs of *Diplectanum aequans*, a parasite of sea bass. Aquacult Int.

[47] Hofrichter R (2004). El mar Mediterráneo. Fauna, Flora, Ecología. I Parte general.

[48] MacLeod CD, Poulin R (2012). Host-parasite interactions: a litmus test for ocean acidification?. Trends Parasitol.

[49] Marcogliese DJ, Cone DK (1996). On the distribution and abundance of eel parasites in Nova Scotia: influence of pH. J Parasitol.

[50] Barker DE, Cone DK (2000). Occurrence of *Ergasilus celestis* (Copepoda) and *Pseudodactylogryrus anguillae* (Monogenea) among wild eels (*Anguilla rostrata*) in relation to stream flow, pH and temperature and recommendations for controlling their transmission among captive eels. Aquaculture.

[51] Buchmann K (1988). Temperature-dependent reproduction and survival of *Pseudodactylogyrus bini* (Monogenea) on the European eel (*Anguilla anguilla*). Parasitol Res.

[52] Lafferty K.D. (1997). Environmental parasitology: What can parasites tell us about human impacts on the environment?. Parasitology Today.

[53] Blanar CA, Munkittrick KR, Houlahan J, MacLatchy DL, Marcogliese DJ (2009). Pollution and parasitism in aquatic animals: a meta-analysis of effect size. Aquat Toxicol.

[54] Riebesell U, Fabry VJ, Hansson L, Gattuso J-P (2011). Guide to best practices for ocean acidification research and data reporting.

[55] Kearn G (1973). An endogenous circadian hatching rhythm in the monogenean skin parasite *Entobdella soleae*, and its relationship to the activity rhythm of the host (*Solea solea*). Parasitology.

[56] Whittington I, Kearn G (1986). Rhythmical hatching and oncomiracidial behaviour in the hexabothriid monogenean *Rajonchocotyle emarginata* from the gills of *Raja* spp. J Mar Biol Assoc UK.

[57] Ernst I, Whittington ID (1996). Hatching rhythms in the capsalid monogeneans *Benedenia lutjani* from the skin and *B. rohdei* from the gills of *Lutjanus carponotatus* at Heron Island, Queensland, Australia. Int J Parasitol.

[58] Kearn GC (1982). Rapid hatching induced by light intensity reduction in the monogenean *Entobdella diadema*. J Parasitol.

[59] Whittington ID, Kearn GC (1989). Rapid hatching induced by light intensity reduction in the polyopisthocotylean monogenean *Plectanocotyle gurnardi* from the gills of gurnards (Triglidae), with observations on the anatomy and behaviour of the oncomiracidium. J Mar Biol Assoc UK.

[60] Ellis EP, Watanabe WO (1993). The effects of hyposalinity on eggs, juveniles and adults of the marine monogenean, *Neobenedenia melleni* treatment of ecto-parasitosis in seawater-cultured tilapia. Aquaculture.

[61] Pavlidis M, Mylonas C (2011). Sparidae: Biology and Aquaculture of Gilthead Sea Bream and Other Species.

[62] Bégout M-L, Lagardére J-P (1995). An acoustic telemetry study of seabream (*Sparus aurata* L.): first results on activity rhythm, effects of environmental variables and space utilization. Hydrobiologia.

[63] Barton BA, Iwama GK (1991). Physiological changes in fish from stress in aquaculture with emphasis on the response and effects of corticosteroids. Annu Rev Fish Dis.

[64] Arends R, Mancera J, Munoz J, Bonga SW, Flik G (1999). The stress response of the gilthead sea bream (*Sparus aurata* L.) to air exposure and confinement. J Endocrinol.

[65] Saraiva A, Costa J, Serrão J, Eiras JC, Cruz C (2015). Study of the gill health status of farmed sea bass (*Dicentrarchus labrax* L., 1758) using different tools. Aquaculture.

[66] Tort L, Rotllant J, Rovira L (1998). Immunological suppression in gilthead sea bream *Sparus aurata* of the North-West Mediterranean at low temperatures. Comp Biochem Phys A.

[67] Hernández A, Tort L (2003). Annual variation of complement, lysozyme and haemagglutinin levels in serum of the gilthead sea bream *Sparus aurata*. Fish Shellfish Immunol.

[68] Antonelli L, Quilichini Y, Marchand B (2010). *Sparicotyle chrysophrii* (Van Beneden and Hesse 1863) (Monogenea: Polyopisthocotylea) parasite of cultured gilthead sea bream *Sparus aurata* (Linnaeus 1758) (Pisces: Teleostei) from Corsica: ecological and morphological study. Parasitol Res.

[69] Marcogliese DJ (2001). Implications of climate change for parasitism of animals in the aquatic environment. Can J Zool.

[70] Marcogliese D (2008). The impact of climate change on the parasites and infectious diseases of aquatic animals. Rev Sci Tech.

[71] Marcogliese DJ (2016). The distribution and abundance of parasites in aquatic ecosystems in a changing climate: more than just temperature. Integr Comp Biol.

[72] Feidantsis K, Pörtner H-O, Antonopoulou E, Michaelidis B (2015). Synergistic effects of acute warming and low pH on cellular stress responses of the gilthead seabream *Sparus aurata*. J Comp Physiol B.

[73] Gandon S, Michalakis Y (2002). Local adaptation, evolutionary potential and host-parasite coevolution: interactions between migration, mutation, population size and generation time. J Evol Biol.

[74] Barber I, Berkhout BW, Ismail Z (2016). Thermal change and the dynamics of multi-host parasite life cycles in aquatic ecosystems. Integr Comp Biol.

