# Supplementary material for: Come rain or come shine: environmental effects on the infective stages of Sparicotyle chrysophrii, a key pathogen in Mediterranean aquaculture
Source: Parasit Vectors. 2018 Oct 25;11:558. doi: 10.1186/s13071-018-3139-3 (PMC6202810; doi:10.1186/s13071-018-3139-3)
Supplement: Supplementary file 1 — Table S1. Parameters of embryonic development of S. chrysophrii by replicate at each temperature. (DOCX 15 kb) [file 13071_2018_3139_MOESM1_ESM.docx]

| Temperature | R | Incubation period (h) | Hatching period (h) | Hatching peak^a^ (h) | Hatching success (%) |
| --- | --- | --- | --- | --- | --- |
| (±0.1ºC) |  | Mean ± SD (range) |  |  |  |
| 10^b^ | R1.1 | 985.3 ± 4.6 (980 ‒ 988) | 8 | 988 | 3.0 |
|  | R1.2 | 1005.0 ± 28.1 (948 ‒ 1044) | 96 | 1020 | 24.0 |
|  | R2.1 | 983.8 ± 7.0 (972 ‒ 988) | 16 | 988 | 17.0 |
| 14 | R1 | 257.8 ± 18.9 (244 ‒ 328) | 84 | 248 | 88.0 |
|  | R2 | 247.1 ± 15.5 (212 ‒ 316) | 104 | 244 | 92.0 |
|  | R3 | 255.3 ± 15.8 (240 ‒ 332) | 83 | 248 | 93.0 |
| 18 | R1 | 178.1 ± 14.5 (148 ‒ 220) | 72 | 172 | 93.0 |
|  | R2 | 178.7 ± 16.1 (160 ‒ 224) | 64 | 176 | 98.0 |
|  | R3 | 177.5 ± 13.7 (160 ‒ 256) | 96 | 176 | 89.0 |
| 22 | R1 | 126.3 ± 5.2 (116 ‒ 152) | 36 | 124 | 89.0 |
|  | R2 | 154.1 ± 22.1 (124 ‒ 200) | 76 | 172 | 95.0 |
|  | R3 | 147.2 ± 25.1 (120 ‒ 192) | 72 | 124 | 97.0 |
| 26 | R1 | 120.2 ± 6.4 (116 ‒ 148) | 32 | 116 | 80.0 |
|  | R2 | 130.1 ± 11.0 (124 ‒ 172) | 48 | 124 | 74.0 |
|  | R3 | 134.0 ± 13.0 (120 ‒ 148) | 28 | 148 | 78.0 |
| 30 | R1 | 142.0 ± 24.5 (124 ‒ 176) | 52 | 124 | 4.0 |
|  | R2 | 148.0 ± 21.9 (128 ‒ 184) | 56 | 128 | 9.0 |
|  | R3 | 153.7 ± 19.3 (124 ‒ 184) | 60 | 148 | 7.0 |

**Additional file 1: Table S1** Parameters of embryonic development of *S. chrysophrii* by replicate at each temperature

^a^Hatching peak, moment when the highest number hatchings was registered

^b^Include replicates with emerged eggs (R=3)
