# Supplementary material for: Come rain or come shine: environmental effects on the infective stages of Sparicotyle chrysophrii, a key pathogen in Mediterranean aquaculture
Source: Parasit Vectors. 2018 Oct 25;11:558. doi: 10.1186/s13071-018-3139-3 (PMC6202810; doi:10.1186/s13071-018-3139-3)
Supplement: Supplementary file 2 — Table S2. Larval longevity and behaviour of S. chrysophrii by replicate at each temperature. (DOCX 15 kb) [file 13071_2018_3139_MOESM2_ESM.docx]

**Additional file 2: Table S2** Larval longevity and behaviour of *S. chrysophrii* by replicate at each temperature

| Temperature | R | N^a^ | Survival period (h) | Swimming ratio (%) |
| --- | --- | --- | --- | --- |
| (±0.1ºC) |  |  | Mean ± SD (range) | Mean ± SD (range) |
| 10^b^ | R1.1 | 3 | 5.3 ± 2.3 (4 ‒ 8) | 33.3 ± 14.4 ( 25 – 50 ) |
|  | R1.2 | 24 | 24.5 ± 23.4 (4 ‒ 96) | 68.6 ± 20.4 (12.5 – 91.7) |
|  | R2.1 | 17 | 51.1 ± 26.8 (4 ‒ 104) | 85.2 ± 16.5 (25.0 – 96.2) |
| 14 | R1 | 88 | 21.2 ± 18.5 (0 ‒ 100) | 61.7 ± 26.0 ( 0 – 92.9 ) |
|  | R2 | 92 | 18.3 ± 16.7 (0 ‒ 88) | 57.7 ± 26.0 (0 – 93.8) |
|  | R3 | 93 | 22.0 ± 18.0 (0 ‒ 84) | 66.3 ± 20.8 (25.0 – 94.7) |
| 18 | R1 | 93 | 19.9 ± 16.7 (0 ‒ 72) | 62.4 ± 26.7 (0 – 93.8) |
|  | R2 | 98 | 17.4 ± 14.2 (0 ‒ 56) | 60.0 ± 27.3 (0 – 92.9) |
|  | R3 | 89 | 14.6 ± 9.3 (0 ‒ 52) | 61.8 ± 21.6 (0 – 92.3) |
| 22 | R1 | 89 | 14.6 ± 9.7 (0 ‒ 52) | 58.7 ± 24.1 (0 – 92.3) |
|  | R2 | 95 | 12.7 ± 8.5 (0 ‒ 48) | 53.8 ± 28.9 (0 – 91.7) |
|  | R3 | 97 | 11.6 ± 8.8 (0 ‒ 44) | 49.1 ± 25.1 (0 – 83.3) |
| 26 | R1 | 80 | 7.9 ± 7.2 (0 ‒ 32) | 37.1 ± 23.9 (0 – 85.7) |
|  | R2 | 74 | 9.8 ± 7.6 (0 ‒ 28) | 43.9 ± 25.7 (0 – 85.7) |
|  | R3 | 78 | 10.7 ± 6.3 (0 ‒ 24) | 50.9 ± 23.6 (0 – 83.3) |
| 30 | R1 | 4 | 4.0 ± 3.3 (0 ‒ 8) | 15.6 ± 12.0 (0 – 25) |
|  | R2 | 9 | 3.6 ± 2.4 (0 ‒ 8) | 18.1 ± 11.0 (0 – 25) |
|  | R3 | 7 | 3.4 ± 1.5 (0 ‒ 4) | 21.4 ± 9.4 (0 – 25) |

^a^N, number of hatched oncomiracidia and used to calculate the mean survival period and swimming ratio ^b^Include replicates with emerged eggs (R=3)
