# Supplementary material for: Come rain or come shine: environmental effects on the infective stages of Sparicotyle chrysophrii, a key pathogen in Mediterranean aquaculture
Source: Parasit Vectors. 2018 Oct 25;11:558. doi: 10.1186/s13071-018-3139-3 (PMC6202810; doi:10.1186/s13071-018-3139-3)
Supplement: Supplementary file 3 — Table S3. Parameters of embryonic development of S. chrysophrii by replicate at each pH level. (DOC 37 kb) [file 13071_2018_3139_MOESM3_ESM.doc]

**Additional file 3: Table S3 Parameters of embryonic development of *S. chrysophrii* by replicate at each pH level**

| pH | R | Incubation period (h) | Hatching period (h) | Hatching peak^a^ (h) | Hatching success (%) |
| --- | --- | --- | --- | --- | --- |
| (± 0.1) |  | Mean ± SD (range) |  |  |  |
| 7.0 | R1 | 152.0 ± 10.5 (144 ‒ 176) | 32 | 148 | 65.0 |
|  | R2 | 146.0 ± 4.9 (144 ‒ 168) | 24 | 144 | 49.0 |
|  | R3 | 151.1 ± 8.9 (144 ‒ 176) | 32 | 148 | 54.0 |
| 7.9 | R1 | 126.3 ± 5.2 (116 ‒ 152) | 36 | 124 | 89.0 |
|  | R2 | 154.1 ± 22.1 (124 ‒ 200) | 76 | 172 | 95.0 |
|  | R3 | 147.2 ± 25.1 (120 ‒ 192) | 72 | 124 | 97.0 |

^a^Hatching peak, moment when the highest number hatchings was registered
