# Supplementary material for: Come rain or come shine: environmental effects on the infective stages of Sparicotyle chrysophrii, a key pathogen in Mediterranean aquaculture
Source: Parasit Vectors. 2018 Oct 25;11:558. doi: 10.1186/s13071-018-3139-3 (PMC6202810; doi:10.1186/s13071-018-3139-3)
Supplement: Supplementary file 4 — Table S4. Larval longevity and behaviour of S. chrysophrii by replicate at each pH level. (DOCX 13 kb) [file 13071_2018_3139_MOESM4_ESM.docx]

**Additional file 4: Table S4** Larval longevity and behaviour of *S. chrysophrii* by replicate at each pH level

| pH | R | N^a^ | Survival period (h) | Swimming ratio (h) |
| --- | --- | --- | --- | --- |
| (± 0.1) |  |  | Mean ± SD (range) | Mean ± SD (range) |
| 7.0 | R1 | 65 | 8.4 ± 8.3 (0 ‒ 40) | 39.4 ± 24.7 (0 – 90.0) |
|  | R2 | 49 | 6.3 ± 4.6 (0 ‒ 20) | 32.8 ± 18.8 (0 – 80.0) |
|  | R3 | 54 | 8.1 ± 7.1 (0 ‒ 32) | 39.8 ± 23.7 (0 – 87.5) |
| 7.9 | R1 | 89 | 14.6 ± 9.7 (0 ‒ 52) | 58.7 ± 24.1 (0 – 92.3) |
|  | R2 | 95 | 12.7 ± 8.5 (0 ‒ 48) | 53.8 ± 28.9 (0 – 91.7) |
|  | R3 | 97 | 11.6 ± 8.8 (0 ‒ 44) | 49.1 ± 25.1 (0 – 83.3) |

^a^N, number of hatched oncomiracidia and used to calculate the mean survival period and swimming ratio
