# Supplementary material for: Come rain or come shine: environmental effects on the infective stages of Sparicotyle chrysophrii, a key pathogen in Mediterranean aquaculture
Source: Parasit Vectors. 2018 Oct 25;11:558. doi: 10.1186/s13071-018-3139-3 (PMC6202810; doi:10.1186/s13071-018-3139-3)
Supplement: Supplementary file 5 — Table S5. Parameters of embryonic development of S. chrysophrii by replicate at each light regime. (DOC 36 kb) [file 13071_2018_3139_MOESM5_ESM.doc]

**Additional file 5: Table S5 Parameters of embryonic development of *S. chrysophrii* by replicate at each light regime**

| Light regime | R | Incubation period (h) | Hatching period (h) | Hatching peak^a^ (h) | Hatching success (%) |
| --- | --- | --- | --- | --- | --- |
| (Light: Darkness) |  | Mean ± SD (range) |  |  |  |
| 12:12 | R1 | 125.2 ± 5.2 (120 ‒ 152) | 32 | 124 | 88.0 |
|  | R2 | 163.6 ± 20.3 (124 ‒ 200) | 76 | 172 | 95.0 |
|  | R3 | 151.8 ± 27.3 (120 ‒ 192) | 72 | 124 | 96.0 |
| 0:24 | R1 | 125.6 ± 5.4 (116 – 140) | 24 | 124 | 93.0 |
|  | R2 | 124.7 ± 4.7 (116 – 140) | 24 | 124 | 87.0 |
|  | R3 | 124.9 ± 18.0 (112 – 232) | 120 | 120 | 88.0 |
| 24:0 | R1 | 132.0 ± 12.2 (108 ‒ 148) | 40 | 120 | 83.0 |
|  | R2 | 140.3 ± 9.9 (108 ‒ 176) | 68 | 140 | 91.0 |
|  | R3 | 152.5 ± 34.6 (120 ‒ 268) | 148 | 148 | 90.0 |

^a^Hatching peak, moment when the highest number hatchings was registered
