# Supplementary material for: Come rain or come shine: environmental effects on the infective stages of Sparicotyle chrysophrii, a key pathogen in Mediterranean aquaculture
Source: Parasit Vectors. 2018 Oct 25;11:558. doi: 10.1186/s13071-018-3139-3 (PMC6202810; doi:10.1186/s13071-018-3139-3)
Supplement: Supplementary file 6 — Table S6. Larval longevity and behaviour of S. chrysophrii by replicate at each light regime. (DOCX 13 kb) [file 13071_2018_3139_MOESM6_ESM.docx]

**Additional file 6: Table S6** Larval longevity and behaviour of *S. chrysophrii* by replicate at each light regime

| Light regime | R | N^a^ | Survival period (h) | Swimming ratio (%) |
| --- | --- | --- | --- | --- |
| (Light: Darkness) |  |  | Mean ± SD (range) | Mean ± SD (range) |
| 12:12 | R1 | 89 | 15.0 ± 10.24 (4 ‒ 52) | 50.3 ± 21.6 (0 – 87.5) |
|  | R2 | 95 | 13.2 ± 7.7 (0 ‒ 48) | 64.6 ± 22.7 (0 – 88.9) |
|  | R3 | 97 | 10.8 ± 7.7 (0 ‒ 40) | 47.9 ± 23.0 (0 – 83.3) |
| 0:24 | R1 | 98 | 10.3 ± 9.2 (4 ‒ 48) | 57.7 ± 23.2 (0 – 90.0) |
|  | R2 | 88 | 9.6 ± 7. 5 (4 ‒ 32) | 56.8 ± 22.7 (0 – 87.5) |
|  | R3 | 82 | 12.2 ± 10.3 (0 ‒ 44) | 56.7 ± 23.7 (0 – 90.0) |
| 24:0 | R1 | 84 | 10.6 ± 5.4 (0 – 32) | 57.3 ± 24.3 (12.5 – 92.3) |
|  | R2 | 86 | 16.2 ± 8.9 (0 ‒ 40) | 65.6± 24.4 (0 – 85.7) |
|  | R3 | 79 | 10.3 ± 6.4 (0 ‒ 32) | 46.2 ± 24.2 (0 – 83.3) |

^a^N, number of hatched oncomiracidia and used to calculate the mean survival period and swimming ratio
