# Supplementary material for: Come rain or come shine: environmental effects on the infective stages of Sparicotyle chrysophrii, a key pathogen in Mediterranean aquaculture
Source: Parasit Vectors. 2018 Oct 25;11:558. doi: 10.1186/s13071-018-3139-3 (PMC6202810; doi:10.1186/s13071-018-3139-3)
Supplement: Supplementary file 7 — Table S7. Parameters of embryonic development of S. chrysophrii by replicate at different salinities and temperatures. (DOC 45 kb) [file 13071_2018_3139_MOESM7_ESM.doc]

**Additional file 7: Table S7 Parameters of embryonic development of *S. chrysophrii* by replicate at different salinities and temperatures**

|  |  | Temperature 18ºC | | | | Temperature 22ºC | | | |
| --- | --- | --- | --- | --- | --- | --- | --- | --- | --- |
| Salinity (ppt) | R | Incubation period (h) | Hatching period (h) | Hatching peak^a^ (h) | Hatching success (%) | Incubation period (h) | Hatching period (h) | Hatching peak^a^ (h) | Hatching success (%) |
|  |  | Mean ± SD (range) |  |  |  | Mean ± SD (range) |  |  |  |
| 27 | R1 | 188.6 ± 18.0 (172 ‒ 220) | 48 | 172 | 86.0 | 137.9 ± 12.3 (124 ‒ 168) | 44 | 148 | 80.0 |
|  | R2 | 193.1 ± 7.6 (176 ‒ 204) | 28 | 196 | 90.0 | 145.5 ± 10.4 (120 ‒ 156) | 36 | 148 | 94.0 |
|  | R3 | 193.4 ± 13.3 (152 ‒ 220) | 68 | 196 | 92.0 | 141.3 ± 10.9 (120 ‒ 152) | 32 | 148 | 82.0 |
| 36 | R1 | 169.6 ± 15.3 (140 ‒ 268) | 128 | 168 | 85.3 | 129.2 ± 9.3 (108 ‒ 156) | 48 | 124 | 74.7 |
|  | R2 | 169.9 ± 10.6 (140 ‒ 200) | 60 | 168 | 82.0 | 126.6 ± 4.9 (124 ‒ 148) | 24 | 124 | 50.0 |
|  | R3 | 167.9 ± 19.2 (144 ‒ 268) | 124 | 168 | 93.0 | 132.8 ± 9.4 (124 ‒ 148) | 24 | 128 | 79.0 |
| 37 | R1 | 165.9 ± 16.5 (148 ‒ 200) | 52 | 148 | 80.0 | 148.5 ± 20.4 (124 ‒ 196) | 72 | 128 | 90.0 |
|  | R2 | 178.7 ± 15.6 (140 ‒ 208) | 68 | 196 | 65.0 | 129.0 ± 7.7 (124 ‒ 148) | 24 | 128 | 62.0 |
|  | R3 | 175.7 ± 20.67 (148 ‒ 228) | 80 | 172 | 49.0 | 134.7 ± 9.9 (124 ‒ 152) | 28 | 128 | 94.0 |
| 38 | R1 | 183.3 ± 12.2 (168 ‒ 204) | 36 | 196 | 82.0 | 138.3 ± 22.7 (124 ‒ 228) | 104 | 148 | 86.0 |
|  | R2 | 182.5 ± 14.2 (168 ‒ 220) | 52 | 172 | 84.0 | 128. 3 ± 8.1 (124 ‒ 148) | 24 | 124 | 60.0 |
|  | R3 | 174.3 ± 10.3 (168 ‒ 196) | 28 | 168 | 96.0 | 136.8 ± 10.3 (128 ‒ 152) | 24 | 128 | 61.0 |
| 47 | R1 | 216.2 ± 15.2 (180 ‒ 244) | 64 | 220 | 86.0 | 182.6 ± 16.6 (148 ‒ 204) | 56 | 192 | 64.0 |
|  | R2 | 220.2 ± 7.7 (196 ‒ 244) | 48 | 220 | 90.0 | 189.6 ± 9.5 (168 ‒ 200) | 32 | 192 | 56.0 |
|  | R3 | 220.3 ± 9.3 (188 ‒ 244) | 56 | 220 | 48.0 | 176.3 ± 6.6 (168 ‒ 196) | 28 | 176 | 74.0 |

^a^Hatching peak, moment when the highest number hatchings was registered
