# Supplementary material for: Come rain or come shine: environmental effects on the infective stages of Sparicotyle chrysophrii, a key pathogen in Mediterranean aquaculture
Source: Parasit Vectors. 2018 Oct 25;11:558. doi: 10.1186/s13071-018-3139-3 (PMC6202810; doi:10.1186/s13071-018-3139-3)
Supplement: Supplementary file 8 — Table S8. Larval longevity and behaviour of S. chrysophrii by replicate at different salinities and temperatures. (DOCX 15 kb) [file 13071_2018_3139_MOESM8_ESM.docx]

**Additional file 8: Table S8** Larval longevity and behaviour of *S. chrysophrii* by replicate at different salinities and temperatures

|  |  | Temperature 18ºC | | | Temperature 22ºC | | |
| --- | --- | --- | --- | --- | --- | --- | --- |
| Salinity | R | N^a^ | Survival period (h) | Swimming ratio (%) | N^a^ | Survival period (h) | Swimming ratio (%) |
| (ppt) |  |  | Mean ± SD (range) | Mean ± SD (range) |  | Mean ± SD (range) | Mean ± SD (range) |
| 27 | R1 | 43 | 14.5 ± 9.0 (0 ‒ 32) | 61.9 ± 21.4 (0 ‒ 87.5) | 40 | 21.5 ± 19.2 (0 ‒ 76) | 55.9 ± 28.2 (0 ‒ 90.9) |
|  | R2 | 45 | 13.4 ± 9.7 (0 ‒ 36) | 56.4 ± 25.6 (0 ‒ 88.9) | 47 | 6.8 ± 4.9 (0 ‒ 16) | 61.9 ± 31.2 (0 ‒ 94.7) |
|  | R3 | 46 | 10.9 ± 6.4 (0 ‒ 32) | 55.2 ± 19.2 (0 ‒ 87.5) | 41 | 12.6 ± 11.4 (0 ‒ 44) | 37.0 ± 25.8 (0 ‒ 75.0) |
| 36 | R1 | 47 | 15.7 ± 8.3 (4 ‒ 32) | 62.8 ± 21.0 (12.5 ‒ 87.5) | 48 | 12.0 ± 4.7 (4 ‒ 28) | 58.4 ± 17.4 (12.5 – 85.7) |
|  | R2 | 40 | 14.7 ± 8.4 (4 ‒ 36) | 59.8 ± 20.8 (12.5 – 87.5) | 25 | 12. 5 ± 5.6 (4 ‒ 28) | 58.9 ± 18.3 (25.0 – 85.7) |
|  | R3 | 52 | 17.0 ± 9.4 (4 ‒ 40) | 64.9 ± 19.6 (25.0 – 90.0) | 39 | 15. 6 ± 8.4 (4 ‒ 48) | 65.7 ± 14.9 (25.0 – 91.7) |
| 37 | R1 | 40 | 29.2 ± 20.4 (4 ‒ 84) | 74.4 ± 26.6 (12.5 – 95.2) | 45 | 14.8 ± 8.5 (4 ‒ 32) | 61.4 ± 24.1 (25.0 – 87.5) |
|  | R2 | 33 | 36.9 ± 17.6 (4 ‒ 64) | 82.2 ± 16.7 (25.0 – 93.8) | 31 | 12.3 ± 5.7 (4 ‒ 28) | 59.0 ± 19.6 (25.0 – 85.7) |
|  | R3 | 25 | 28.3 ± 18.1 (4 ‒ 68) | 75.4 ± 22.0 (25.0 – 93.3) | 47 | 14.1 ± 5.9 (4 ‒ 32) | 63.4 ± 15.9 (25.0 – 87.5) |
| 38 | R1 | 41 | 30.0 ± 11.9 (4 ‒ 56) | 79.4 ± 13.9 (25.0 – 92.9) | 43 | 14.7 ± 11.7 (4 ‒ 44) | 53.4 ± 25.3 (25.0 – 90.9) |
|  | R2 | 33 | 25.9 ± 13.2 (4 ‒ 52) | 76.3 ± 18.4 (25.0 – 92.3) | 30 | 10.9 ± 9.3 (4 ‒ 32) | 46.8 ± 26.5 (25.0 – 87.5) |
|  | R3 | 48 | 23.0 ± 11.7 (4 ‒ 52) | 74.1 ± 17.4 (12.5 – 92.3) | 30 | 13.2 ± 10.1 (4 ‒ 40) | 54.7 ± 24.9 (25.0 – 90.0) |
| 47 | R1 | 43 | 13.5 ± 8.9 (0 ‒ 36) | 59.4 ± 28.3 (0 ‒ 88.9) | 32 | 15. 8 ± 14.8 (0 ‒ 44) | 51.2 ± 32.9 (0 ‒ 90.9) |
|  | R2 | 45 | 13.1 ± 10.2 (0 ‒ 44) | 54.5 ± 28.5 (0 ‒ 90.9) | 28 | 17.7 ± 13.8 (0 ‒ 44) | 59.6 ± 32.2 (0 ‒ 90.9) |
|  | R3 | 24 | 13.7 ± 9.5 (0 ‒ 36) | 57.7 ± 28.6 (0 ‒ 88.9) | 37 | 13.9 ± 13.1 (0 ‒ 44) | 50.4 ± 30.0 (0 ‒ 90.0) |

^a^N, number of hatched oncomiracidia and used to calculate the mean survival period and swimming ratio
